# Analyses of a whole-genome inter-clade recombination map of hepatitis delta virus suggest a host polymerase-driven and viral RNA structure-promoted template-switching mechanism for viral RNA recombination

**DOI:** 10.18632/oncotarget.18339

**Published:** 2017-06-01

**Authors:** Mei Chao, Tzu-Chi Wang, Chia-Chi Lin, Robert Yung-Liang Wang, Wen-Bin Lin, Shang-En Lee, Ying-Yu Cheng, Chau-Ting Yeh, Shan-Bei Iang

**Affiliations:** ^1^ Department of Microbiology and Immunology, Chang Gung University, Guishan, Taoyang, Taiwan; ^2^ Division of Microbiology, Graduate Institute of Biomedical Sciences, Chang Gung University, Guishan, Taoyang, Taiwan; ^3^ Department of Hepato-Gastroenterology, Liver Research Center, Chang Gung Memorial Hospital, Guishan, Taoyang, Taiwan; ^4^ Department of Biomedical Sciences, Chang Gung University, Guishan, Taoyang, Taiwan

**Keywords:** hepatitis delta virus, mammalian RNA polymerase, ribozyme, RNA recombination, template-switching, Immunology and Microbiology Section, Immune response, Immunity

## Abstract

The genome of hepatitis delta virus (HDV) is a 1.7-kb single-stranded circular RNA that folds into an unbranched rod-like structure and has ribozyme activity. HDV redirects host RNA polymerase(s) (RNAP) to perform viral RNA-directed RNA transcription. RNA recombination is known to contribute to the genetic heterogeneity of HDV, but its molecular mechanism is poorly understood. Here, we established a whole-genome HDV-1/HDV-4 recombination map using two cloned sequences coexisting in cultured cells. Our functional analyses of the resulting chimeric delta antigens (the only viral-encoded protein) and recombinant genomes provide insights into how recombination promotes the genotypic and phenotypic diversity of HDV. Our examination of crossover distribution and subsequent mutagenesis analyses demonstrated that ribozyme activity on HDV genome, which is required for viral replication, also contributes to the generation of an inter-clade junction. These data provide circumstantial evidence supporting our contention that HDV RNA recombination occurs via a replication-dependent mechanism. Furthermore, we identify an intrinsic asymmetric bulge on the HDV genome, which appears to promote recombination events in the vicinity. We therefore propose a mammalian RNAP-driven and viral-RNA-structure-promoted template-switching mechanism for HDV genetic recombination. The present findings improve our understanding of the capacities of the host RNAP beyond typical DNA-directed transcription.

## INTRODUCTION

RNA-directed RNA transcription is carried out by most RNA viruses using the viral-encoded RNA-dependent RNA polymerase (RdRp). However, host-encoded RNA polymerases (RNAPs), which are typically DNA-directed, can also perform RNA-directed RNA transcription of human hepatitis delta virus (HDV) in the nucleus [1, 2]. HDV is a subviral satellite of hepatitis B virus (HBV), which provides envelope proteins for HDV assembly and transmission [3, 4]. HDV has a small (approximately 1.7-kb) genome of single-stranded RNA. The HDV genome and its complementary RNA species (known as the antigenome) are unlike those of any other RNA virus in terms of having a circular conformation, folding into an unbranched rod-like structure via intra-molecular base-pairing, and containing pseudoknot ribozymes [5, 6, 7, 8, 9]. *In vivo*, HDV RNA replication is considered to involve a double rolling-circle mechanism in which the genomic and antigenomic RNAs are synthesized as multimers and can be processed to unit-length circular RNAs [1, 2]. This processing involves ribozyme-mediated site-specific cleavage and ligation, possibly via a host ligase [10]. HDV has a third subgenomic RNA species (∼0.8-kb) of antigenomic polarity that has a 5’-cap and 3’-poly(A) tail and encodes its only viral protein, the delta antigen (HDAg). HDAg is a basic oligomeric protein that interacts with the unbranched rod-like structure of both genomic and antigenomic HDV RNAs [11] and transports the HDV RNA to the nucleus [12]. HDAg occurs as both small (S-HDAg) and large (L-HDAg) forms. During HDV replication, host adenosine deaminase acting on RNA 1 (ADAR1) targets the antigenomic RNA, converting the adenosine that corresponds to the amber termination codon of S-HDAg to an inosine. This triggers the production of an mRNA in which the UAG amber termination codon for S-HDAg instead encodes tryptophan (UGG). Consequently, this so-called amber/W RNA editing leads to the production of L-HDAg, which has an additional 19-20 amino acids (aa) at the C-terminus compared to S-HDAg [13]. The two forms have distinct functions: S-HDAg is required for RNA replication, while L-HDAg inhibits replication and is crucial for packaging [14, 15, 16].

The evolution rate of HDV is similar to those of other animal RNA viruses that encode their own RdRps [17]. The lack of proofreading activity of host RNAP on the atypical HDV RNA template might contribute to the high mutation rate of HDV. There are the comparisons between different natural isolates and eight major clades of HDV have been reported to date (HDV-1∼8), which are characterized by as much as 40% sequence divergence [18]. In addition to polymerase incorporation errors and ADAR1-catalyzed amber/W editing, RNA recombination is also known to contribute to the genetic heterogeneity of HDV [19, 20]. This process contributes not only to the sequence variability of a virus but also to repair damaged viral genomes and maintain the infectivity of RNA viruses [21]. The replicating template-switching mechanism appears to be the most common mechanism for homologous RNA recombination in many RNA viruses [22, 23]. The results from experimental studies have suggested that the host RNAP can initiate HDV replication from transfected in-vitro-transcribed linear HDV RNA species [24, 25, 26]. Moreover, traditional homologous RNA recombination involving two replicating HDV RNAs has also been observed in natural HDV mixed-clade infections and in cultured cells co-transfected with two HDV sequences of the same or different clades [20, 27, 28]. Recently, naturally occurring inter-clade HDV-1/HDV-2 and inter-subtype HDV-4/HDV-4M recombinants were reported in Vietnam and the Miyako Islands, Japan, respectively, as assessed using phylogenetic approaches [29, 30].

Since the molecular mechanism underlying homologous HDV RNA recombination remains unclear, we set out to identify the factors responsible for promoting host RNA template-switching on HDV RNA genome. We established a whole-genome HDV-1/HDV-4 recombination map using cloned HDV sequences co-transfected into cultured cells. The recombinants were analyzed by restriction fragment length polymorphism (RFLP) of 400 cloned reverse transcription (RT)-PCR products. Subsequent sequencing analyses indicated at least 54 homologous recombinants with 57 crossovers mapping to 22 junctions were identified. We evaluated the distributions of crossovers on the map to gain new insights into not only the molecular mechanism underlying HDV RNA recombination, but also the related mechanisms of HDV virology, such as clade-specific complementation of HDV RNA replication by S-HDAg between HDV-1 and HDV-4. Our functional studies of chimeric S-HDAg further demonstrated that the 100 aa at the N-terminus are important for such clade-specific complementation of RNA replication. Mutagenesis data further suggested that template-switching occurs frequently during the synthesis of genomic HDV RNA and that the ribozyme activity and a RNA bulge present in the characteristic HDV rod-like structure involved in the generation of inter-clade junctions. Finally, we propose a model that explains the roles of the host RNAP and viral RNA structures in facilitating a form of HDV RNA recombination capable of producing new HDV sequences that have correct genome structures and encode functional viral proteins.

## RESULTS

### Inter-clade recombination map of HDV

**Figure 1:**
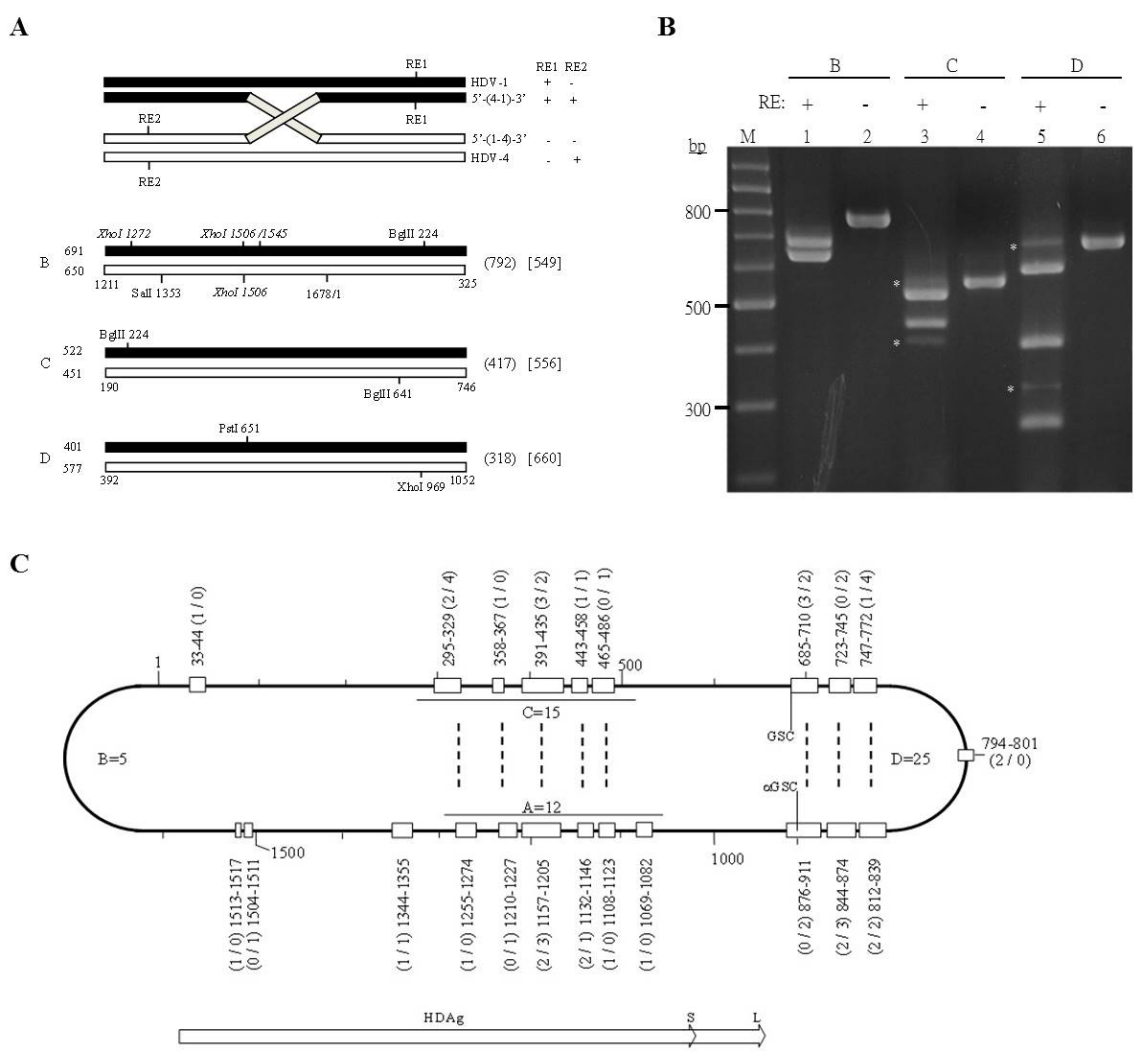
Detection of HDV-1/HDV-4 recombination by RT-PCR-RFLP **A.** Schematic diagram of the amplified HDV cDNAs and RFLP patterns used to detect HDV RNA recombinants. The HDV-1 and HDV-4 sequences are indicated as closed and open bars, respectively. Top: RE1 and RE2 represent the REs used in the RFLP assay. The genomic organizations and RFLP profiles of HDV-related species are summarized on the right. Bottom: Restriction maps of the PCR products. The cutting sites for the RE used in the RFLP assays for fragments B (SalI/BglII), C (BglII), and D (PstI/XhoI) are indicated. The predicted sizes of the largest digested bands for the parental HDV-1 and HDV-4 sequences are summarized on the left. Numbers given in parentheses and square brackets on the right indicate the sizes of the largest digested bands for the potential 5’-(1-4)-3’ and 5’-(4-1)-3’ recombinants, respectively. **B.** The RE-cleaved PCR products of the HDV genomes were electrophoresed on a 3% agarose gel and stained with ethidium bromide. Lanes: M, 100-bp ladder molecular size markers; 2, 4, and 6, undigested PCR products of fragments B, C, and D, respectively; 1, 3, and 5, fragments B, C, and D digested with SalI/BglII, BglII, and PstI/XhoI, respectively. The potential recombinant bands are indicated with stars. **C.** Schematic diagram of whole-genome HDV-1/HDV-4 recombination map. The HDV rod-like RNA genome is represented by the bold line. The open boxes represent the identified crossovers. The nt numbers and clone numbers of the sequenced recombinants are shown outside the HDV rod. The numbers given in parentheses before and after the slash (/) represent the clone numbers for the 5’-(1-4)-3’ and 5’-(4-1)-3’ recombinants, respectively. Twelve [34], five, 15, and 25 recombination events were obtained from fragment A∼D., respectively. Dotted lines indicate eight pairs of crossovers each located exactly opposite one another on the HDV rod-like structure. The abbreviations “GSC” and “GSC” represent the genomic (nt 685/686) and antigenomic (nt 900/901) self-cleavage sites, respectively [8, 9]. The white arrows indicate the ORFs for S- and L-HDAg.

At present, replication-competent cDNA clones are available for HDV-1∼4 [16, 31, 32, 33]. These serve as excellent experimental tools for investigating HDV RNA recombination in cultured cells co-transfected with two HDV sequences. HDV RNA recombination of a region corresponding to nt 886-1308 (designated as fragment A in this study) was previously analyzed by XhoI-RFLP and sequencing of cloned PCR products in cells co-transfected with HDV-1 and HDV-4 RNAs, and the recombination frequency was found to be as high as 12% [34]. To evaluate the biological significance of genetic recombination in HDV, we herein established a whole-genome HDV-1/HDV-4 recombination map using the same RNA samples. Three additional partially overlapping fragments were RT-PCR amplified [fragments B (nt 1211-325), C (nt 190-746), and D (nt 392-1052), the four together covering the whole genome] and subjected to RFLP assays (Figure 1). The REs used in the RFLP assays and the predicted RFLP profiles (SalI-BglII-RFLP, BglII-RFLP, and PstI-XhoI-RFLP for fragments B∼D, respectively) are summarized in Figure 1A. The products obtained from the RT-PCR-RFLP assays were separated on a 3% agarose gel and stained with ethidium bromide (Figure 1B). If one (or an odd number of) recombination event(s) occurred between two RE cutting sites, the electrophoretic mobilities of the bands corresponding to parental and recombinant sequences could be distinguished and complex RFLP patterns might be observed (Figure 1B). Indeed, such patterns were observed for fragments C and D (Figure 1B, lanes 3 and 5), while only the bands corresponding to the parental RNAs were readily observed for fragment B (lane 1).

The obtained PCR products were cloned into T-vectors. DNAs extracted from 100 colonies were subjected to RFLP assays to screen for the HDV recombinants. Thirteen and 21 recombinants were identified using BglII-RFLP and PstI-XhoI-RFLP for fragments C and D, respectively. Two RFLP assays, specifically XhoI-RFLP and SalI-BglII-RFLP, were employed to detect the potential HDV recombinants for fragment B (Figure 1A). Although the PCR-SalI-BglII-RFLP bands corresponding to the potential HDV recombinants for fragment B were not readily visible on stained agarose gels (Figure 1B, lane 1), three and two recombinants were detected by SalI-BglII-RFLP and XhoI-RFLP assays of 100 cloned PCR products, respectively. In total, 51 recombinants, including the 12 previously identified for fragment A [34], were detected from 400 clones using five RFLP analyses. To further evaluate the genomic organizations of the newly identified HDV recombinants, all of the potential recombinant clones and 20 clones of each parental HDV-1 and HDV-4 candidate were subjected to sequence analysis. For the tested clones of fragments A [34] and B, the sequencing data were in complete agreement with the RFLP results. In contrast, one clone for fragment C and two clones for fragment D, originally identified as parental sequences in PCR-RFLP assays, were found to be recombinants with two inter-clade junctions between the two RE sites, located at nt 295-329/391-435, nt 685-710/747-772, and nt 812-839/876-911, respectively. Therefore, sequencing of 211 cloned PCR products identified at least 54 recombinants (57 recombination events) with 22 different inter-clade junctions (Figure 1C). Sequence alignment indicated that all of the identified crossovers were located precisely (i.e., with no deletion, insertion, or point mutation) at regions that were homologous between the two parental sequences (Supplementary Figure 1). These data are consistent with the notion that HDV RNA recombination is homologous.

### Trans-activation activities of the chimeric S-HDAgs

**Figure 2:**
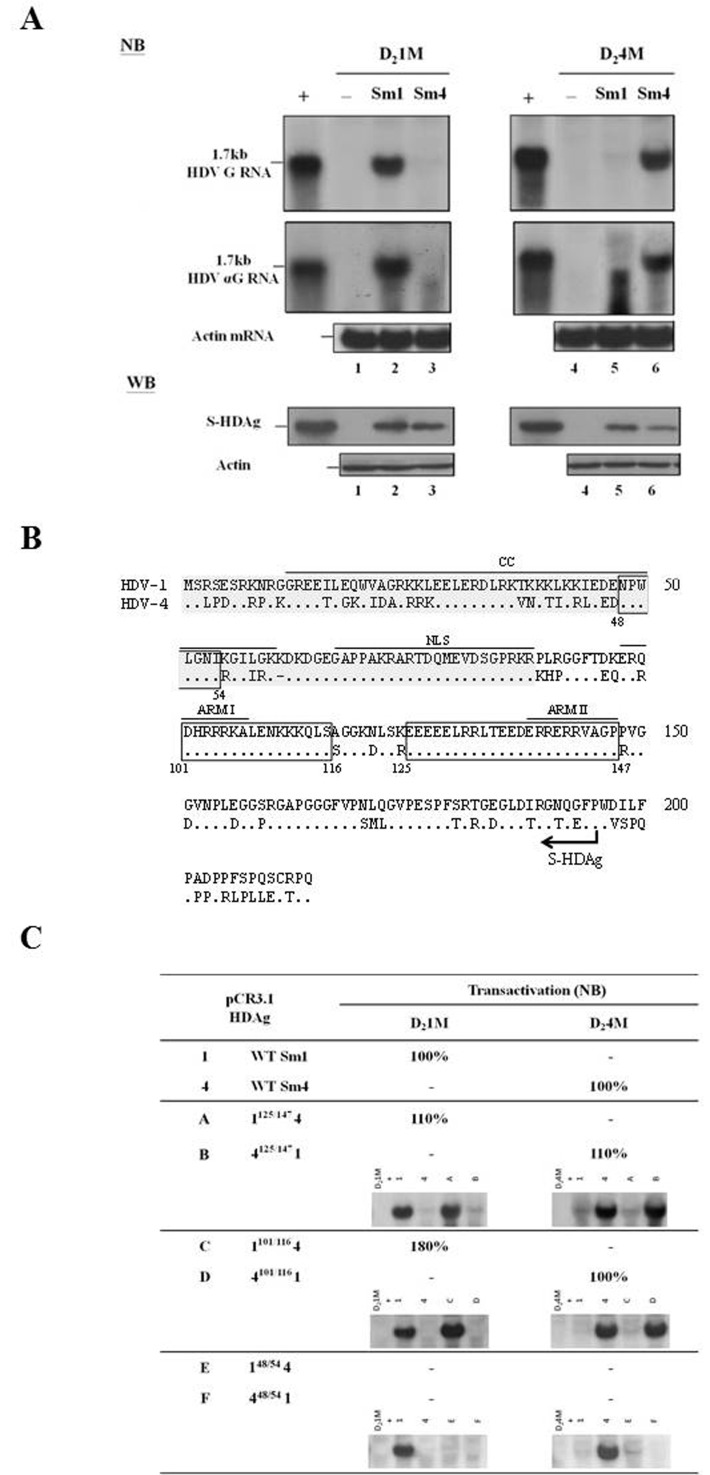
Trans-activation abilities of WT HDV-1 and HDV-4 S-HDAg **A.** NB and WB analyses of HDV RNA replication and HDAg expression, respectively. COS7 cells were co-transfected with the HDV mutant genome-encoding vectors, pSVL-D_2_IM (lanes 1-3) or pSVL-D_2_4M (lanes 4-6) plus empty plasmid (lanes 1 and 4) or plasmids encoding the S-HDAg of HDV-1 (lanes 2 and 5) or HDV-4 (lanes 3 and 6). NB: Lane + contains RNA extracted from cells transfected with an HDV genome-expressing plasmid. RNA loading was monitored by probing for the actin mRNA. WB: Lane + contains protein samples extracted from a cell line stably expressing S-HDAg. Actin protein levels were determined and used to compare the total protein levels in each lane. **B.** Sequence comparison of the HDV-1 and HDV-4 HDAg. The dots represent identical aa sequences, and the three homologous regions that served as the junctions for the S-HDAg chimeras are boxed. Functional domains identified in other studies are indicated. Two models for RNA binding of HDAg [the widely cited ARM (arginine-rich motifs ARM I and II) model [37] and the N-terminal 88 aa RBD (RNA binding domain; shaded gray) model [35]] are shown. The coiled-coil domain (CC) involved in dimerization and oligomerization and NLS (nuclear localization signal) [36, 38] are also indicated. **C.** The relative levels of RNA replication supported by the chimeric HDAg constructs. Cells were co-transfected with pSVL-D_2_1M or pSVL-D_2_4M plus the various HDAg expression vectors. HDV RNA replication was analyzed by NB. The values shown are given relative to the RNA expression level of the homologous WT HDAg, as averaged from two independent experiments. Relative activities that were less than 5% of the WT are indicated with a minus sign. The representative NB data were also shown.

Studies of HDV RNA recombination have helped us better elucidate the molecular regulation of the HDV life cycle [28]. As shown in Figure 1C, we identified 16 recombination events in the ORF encoding HDAg. To further elucidate the phenotypic consequences of the resulting recombinant HDAgs, we analyzed the trans-activation abilities of the parental and chimeric S-HDAgs. Notably, although a replication-competent clones of HDV-1 and HDV-4 have been published [33], the trans-activation activities of the HDV-1 and HDV-4 S-HDAgs have not been simultaneously examined. Here, cells were co-transfected with vectors encoding wild-type (WT) HDV-1 and HDV-4 S-HDAg plus plasmids expressing HDV-1 or HDV-4 genomic RNA defective for HDAg production. Six days after transfection, total RNA was harvested and HDV RNA was analyzed by Northern blotting (NB). Similar results were obtained when detecting genomic and antigenomic RNA (Figure 2A). The expression of the various S-HDAgs was confirmed by Western blotting (WB) (Figure 2A). As expected, cells transfected with constructs encoding HDAg-defective HDV-1 or HDV-4 alone showed no evidence of HDV RNA replication (Figure 2A, lanes 1 and 4). Interestingly, HDV replication could be rescued by S-HDAg expression, but only by the S-HDAg of the same clade (lanes 2 and 3 for HDV-1 RNA replication; lanes 5 and 6 for HDV-4 RNA replication). This suggests that the ability of the S-HDAg of HDV-1 and HDV-4 to support replication of HDAg-defective RNA is clade-specific.

We performed aa sequence alignment between the HDAgs of HDV-1 and HDV-4 (Figure 2B), and then used domain swapping between HDV-1 and HDV-4 clones to create a series of chimeric S-HDAg expression constructs. The HDAg coding region was divided into two domains based on the presence or absence of recombination junctions, as shown in Figure 1C. The recombination junctions mapping to nt 1157-1205 and nt 1255-1274 (Figure 1C), which corresponded to the conserved regions in the S-HDAg ORF spanning aa 125-147 and aa 101-116, respectively, were used to construct four plasmids expressing HDAg chimeras. The N-termini of Sm-1^125/147^4 and Sm-4^125/147^1 corresponded to HDV-1 and HDV-4 sequences, respectively. Similarly, the crossover at nt 1255-1274 yielded the chimeric HDAgs, Sm-1^101/116^4 and Sm-4^101/116^1. Plasmids expressing Sm-1^48/54^4 and Sm-4^48/54^1 chimeras, which used a junction that was not identified in the map, were also constructed. The expression of various chimeric HDAgs determined by WB was summarized in Supplementary Figure 2. The abilities of these six S-HDAg chimeras to trans-activate RNA replication of HDV-1 and HDV-4 were examined by NB analysis. As summarized in Figure 2C, Sm-1^125/147^4 and Sm-1^101/116^4 supported RNA replication from HDV-1 but not HDV-4, whereas Sm-4^125/147^1 and Sm-4^101/116^1 trans-activated RNA synthesis from HDV-4 but not HDV-1, and Sm-1^48/54^4 and Sm-4^48/54^1 failed to support RNA replication from either HDV-1 or HDV-4. Our data further suggest that selection of genetic recombinants might occur during HDV replication and that the N-terminal-most 100 aa of HDAg, which encode important RNA binding and dimerization domains [35, 36] (Figure 2B), were responsible for the clade-specific trans-activation of HDV-1 and HDV-4 RNA replication.

### Replication competence of the recombinant HDV genomes

**Figure 3:**
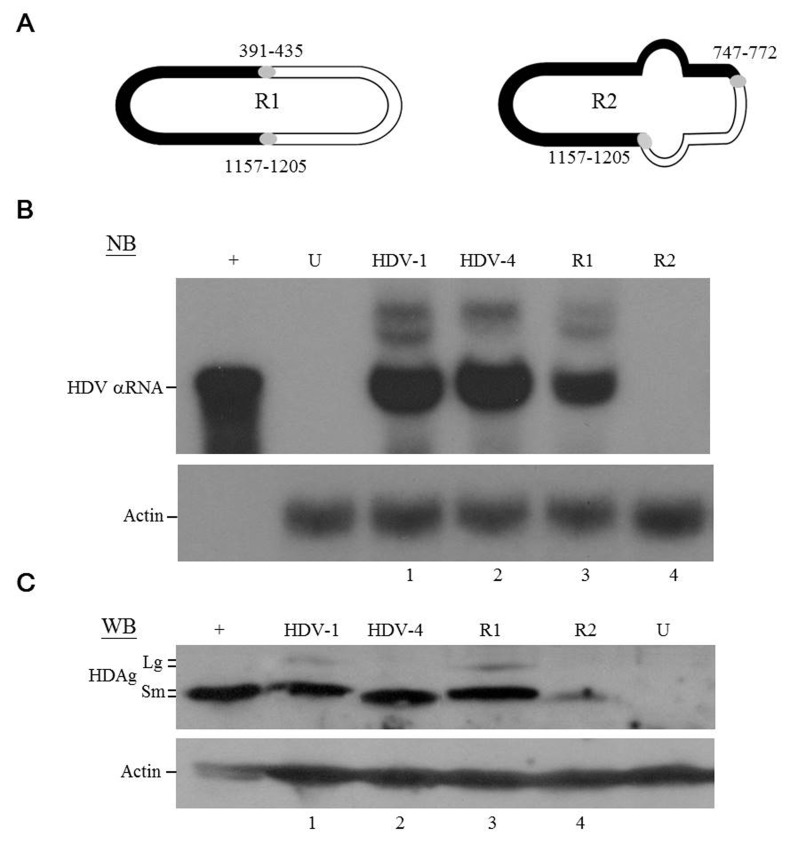
HDV replication and HDAg expression of recombinant HDV **A.** Schematic diagram of the circular HDV recombinant genomes. The HDV-1 and HDV-4 sequences are indicated by closed and open regions, respectively. The segment with a low degree of intra-molecular base-pairing in recombinant R2 is depicted as a bulb. The inter-clade junctions are indicated by gray circles. **B.** NB and **C.** WB analyses of HDV RNA replication and HDAg expression. Lanes: 1∼4, samples extracted from COS7 cells transfected with plasmids expressing WT HDV-1, WT HDV-4, recombinant R1, and recombinant R2 RNAs, respectively. Lanes: +, *in vitro* transcribed HDV RNA for NB and protein extracted from cells stably expressing S-HDAg for WB; and U, RNA or protein samples extracted from untransfected cells.

Interestingly, a close examination of the distribution of crossovers (Figure 1C) revealed that eight pairs (those at nt 295-329/1255-1274, 358-367/1210-1227, 391-435/1157-1205, 443-458/1132-1146, 465-486/1108-1123, 685-710/876-911, 723-745/844-874, and 747-772/812-839) were each located exactly opposite one another on the HDV rod-like structure. The fact that most of the recombination events could find their partners on the opposite side of the HDV rod-like RNA genome (Figure 1C) strongly suggested selection favored replication-competent HDV recombinants with correct unbranched rod-like structure. We thereby constructed two recombinant HDV genomes: each had two inter-clade junctions, but one had a rod-like structure whereas the other did not. Since recombination was frequently observed at nt 1157-1205 (Figure 1C) and the resulting chimeric HDAgs (with a junction mapped to aa 125-147) were functional (Figure 2C), nt 1157-1205 was used as one of the junctions in the recombinant genomes. Two recombination “hot spots”, nt 391-435 and nt 747-772 (Figure 1C), were chosen as the second junctions for the R1 and R2 recombinant genomes, respectively. Since nt 391-435 was opposite nt 1157-1205 whereas nt 747-772 was not, the rod-like structure was maintained for R1 but not R2 (Figure 3A and Supplementary Figure 3). The HDV RNA and HDAg were analyzed using NB and WB, respectively (Figures 3B and 3C), at 6 days post-transfection, respectively. We found that WT HDV-1 and -4 replicated equally well in this system (Figure 3B, lanes 1 and 2), which was consistent with a previous report [33]. We further observed that the R1 genome replicated at about 65% of the WT level (Figure 3B, lane 3), whereas the R2 genome failed to replicate (Figure 3B, lane 4). These data suggest that the rod-like structure is strictly required for genomic replication. Interestingly, R1 expressed HDAg at a level comparable to that of WT (Figure 3C, lanes 1 and 3), and R2, although it failed to undergo RNA replication, expressed HDAg to 15% of the WT level (Figure 3C, lane 4). Taken together, our results indicate that the emergence of replication-competent recombinant HDV genomes with correct unbranched rod-like structures and functional HDAg might contribute to generating new HDV strains.

### A genomic self-cleavage site serves as an inter-clade junction

**Figure 4:**
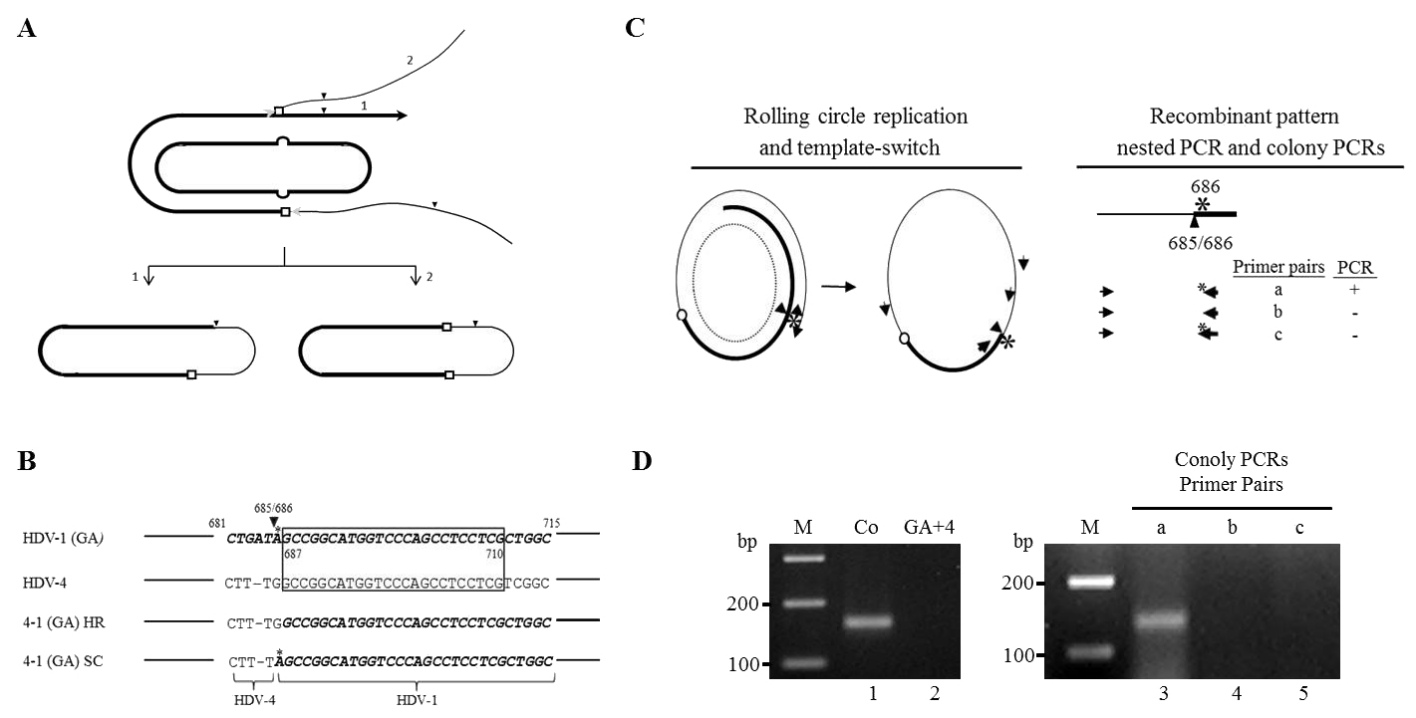
Involvement of ribozyme activity in generating the inter-clade junction of HDV recombinants **A.** Schematic description of the generation of an inter-clade junction. The thick and thin lines represent two HDV sequences. Line with black arrowhead signifies the newly synthesized nascent RNA. The gray and black arrowheads symbolize the template-switching of HDV RNAs by host RNAP and self-cleavage site, respectively. Homologous recombination sites between two RNAs are depicted as open squares. The individual steps are described in the text. **B.** Alignment of nucleic acid sequences (nt 681-715) from HDV-1 GA mutant and HDV-4 sequences. The HDV-1 sequences are in bolded italics. The homologous region (nt 687-710) between the HDV-1 GA mutant and HDV-4 is boxed. HR and SC indicate the 5’-(4-1)-3’ recombinant sequences resulting from a homologous recombination event at nt 687-710 and a self-cleavage/ligation reaction at 685/686, respectively. The sequence variation between recombinants HR and SC in the region covering nt 681-715 is limited to a single nt: nt 686. **C.** Left: Schematic depiction of the generation of HDV recombinants generated via RNA replication, template-switching (symbolized with a small circle), and self-cleavage/ligation (represented as an arrowhead). The dashed oval and asterisk symbolize the template used for genomic replication and the G-to-A mutation at nt 686, respectively. The thick and thin lines represent HDV-1 GA and HDV-4 sequences, respectively. Lines with arrowheads signify the newly synthesized nascent RNA. The individual steps are described in the text. The locations of the two primer pairs used in the nested PCR reactions are shown outside and inside of the HDV circular genome. The thick and thin lines with arrowheads represent primers for the HDV-1 GA and HDV-4 sequences, respectively. Right: Molecular natures of the recombinant patterns. Cloned PCR products obtained from the nested PCR reactions (top) were subjected to colony PCRs using three primer pairs (a, b, and c). HDV recombinants with an inter-clade junction generated by self-cleavage/ligation could be detected by primer pair a only. **D.** The PCR products were subjected to 3% agarose gel electrophoresis and stained with ethidium bromide. Lanes: M, 100-bp ladder molecular size markers; 1, nested PCR products amplified from RNA of co-transfected cells; 2, nested PCR products amplified from RNA of a mixture of separately transfected cells. Colony PCR-based differentiation of various 5’-(4-1)-3’ recombinant patterns in a region containing a genomic self-cleavage site were shown in lanes 3-11. Lanes 3-5 show representative results for recombinants of interest.

We have previously proposed a replicating template-switching model for HDV RNA recombination [39]. If a recombinant HDV genome is generated by only one or an odd number of recombination events, then it follows that the self-cleavage site must serve as an inter-clade junction to maintain the circular conformation of the HDV genome (Figure 4A, step 1). Conversely, if another recombination event occurs prior to the appearance of the self-cleavage site, both inter-clade junctions will represent real recombination sites (Figure 4A, step 2). We hypothesized that the identification of a self-cleavage site as an inter-clade junction would provide substantial evidence that HDV RNA recombination occurs via a replicating mechanism involving viral ribozyme activity and host RNAP. Indeed, recombination occurred frequently in a region (nt 685-710) containing a genomic self-cleavage site (nt 685/686) [8, 9] (Figure 1C). However, the genomic self-cleavage site (nt 685/686) is located within a long stretch (nt 685-710) that is homologous between HDV-1 and HDV-4. To differentiate whether the crossover at nt 685-710 arose via self-cleavage/ligation or homologous recombination, we employed a published HDV-1 replication-competent mutant carrying a G-to-A mutation at nt 686 (GA mutant, located immediately 3’ of the genomic self-cleavage site) [40]. In this system, the HDV 5’-(4-1)-3’ recombinants that arose from self-cleavage/ligation or real template-switching at the nearby homologous region could be differentiated by the nt at position 686 (A or G, respectively) (Figure 4B). As summarized in Figure 4C (left), if genomic RNA replication is initiated on the HDV-1 GA RNA and a recombination event occurs on the opposite side of the genomic self-cleavage site, a 5’-(1-4)-3’ junction is generated. Replication proceeds until the genomic ribozyme domain is exposed. Self-cleavage/ligation then generates a 5’-(4-1)-3’ junction. The detection of this recombination pattern would support the hypothesis that HDV RNA recombination occurs via a replicating mechanism. The HDV-1 GA mutant was co-transfected or separately transfected into cultured cells along with WT HDV-4. Six days post-transfection, total cellular RNAs were extracted. Cellular RNAs were then amplified by a nested PCR reaction designed to specifically amplify 5’-(4-1)-3’recombinants carrying an inter-clade junction at or near the genomic self-cleavage site (Figure 4C, left). A PCR product of the expected size was observed in RNA extracted from co-transfected cells (Figure 4D, lane 1) but not from the mixture of separately transfected cells (Figure 4D, lane 2). The PCR product (Figure 4D, lane 1) was then cloned into a T-vector, and 70 colonies containing HDV cDNA inserts were subjected to three PCR reactions using primer pairs a-c (Figure 4C, right). The HDV-4-specific forward primer was used in all three PCR reactions. The reverse primers (nt 705-686) used for reactions a and b differed only in the presence of an A or G residue, respectively, at nt 686. The reverse primer for reaction c was the HDV-1-specific primer for which the 3’ end had been extended to nt 683. The recombinants of interest could be amplified by primer pair a but not by primer pairs b and c. Of the 70 picked colonies, three clones were found to represent recombinant pattern which corresponded to the presence of a mutated A residue at nt 686 and sequences upstream and downstream of the genomic self-cleavage site derived from different HDV clades. These three clones were further confirmed by sequencing. Our data clearly demonstrated that the inter-clade junction of HDV recombinants could be generated by self-cleavage/ligation, which is a key step in the host RNAP-driven HDV RNA replication. Thus HDV RNA recombination appears to be carried out by a replication-dependent template-switching mechanism that involves the host RNAP.

### A bulge on the HDV rod-like RNA promotes RNA recombination

**Figure 5:**
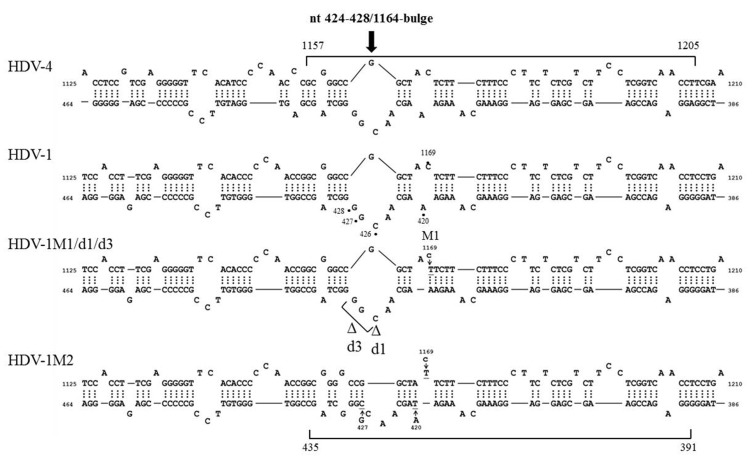
The HDV RNA structure affects HDV RNA recombination HDV rod-like genome structures formed by HDV-1 and HDV-4 between nt 386-464 and nt 1125-1210. The position of the nt 424-428/1164-bulge and the recombination junction (nt 1157-1205) are shown. The positions of the mutation sites and the resulting structural variations for HDV-1 are indicated.

We failed to find any consensus sequence within or around the HDV recombination junctions (Supplementary Figure 1), indicating that HDV recombination was not guided by a sequence-specific mechanism. We further observed that HDV recombination occurred at multiple regions on the HDV rod-like RNA, and showed different frequencies at these regions (Figure 1C). We focused on the identification of the potential RNA structure promoting recombination at nt 1157-1205, which was a “hot spot” observed in patients and in co-transfected cultured cells (Figure 1C) [20, 27, 28]. To try to narrow down this relatively long homologous region, we first dissected it into two smaller homologous regions by the introduction of a C-to-T mutation at nt 1169 (Figure 5). This generated HDV-1 mutant M1 did not change the aa sequence of HDAg. The regions homologous between HDV-1 mutant M1 and HDV-4 were found at nt 1157-1168 and nt 1170-1205. Cells were co-transfected with HDV-1 M1 mutant/HDV-4 genome expressing plasmids, RNA was extracted at 6 days post-transfection, and RT-PCR reactions were performed using two clade-specific primer pairs (which amplified a region covering nt 1071-1269) to specifically amplify 5’-(1-4)-3’ and 5’-(4-1)-3’ recombinants, respectively. Fifteen cloned HDV plasmids representing each kind of HDV recombinant were subjected to SacII/BstBI-RFLP assays. As summarized in Table 1 (left column), the cutting sites for SacII and BstBI are absent in HDV-1 but present in HDV-4 at nt 1155 and 1205, respectively. Based on the RFLP patterns, the molecular nature of the recombinants could be elucidated. As summarized in Table 1, the crossover site of 66.7% of the recombinants from two independent co-transfections could be mapped to nt 1155-1205. Sequence analyses further indicated that 90.0% and 93.8% (in the two independent experiments) of these recombinants carried a crossover mapped to nt 1157-1168. Accordingly, we searched for potential secondary structures around nt 1157-1168, and found that an asymmetric bulge was located between nt 424-428 and nt 1164 (designated the nt 424-428/1164-bulge) on the HDV rod-like genome and antigenome (the genomic RNA structure is presented in Figure 5). To investigate the role of this bulge in HDV RNA recombination, we constructed three different mutants designed to reduce or eliminate this specific bulge on the HDV-1 RNA: one involved further mutation of the HDV-1 M1 mutant to specifically alter nt 420 and 427 (M2); one harbored a mutation at nt 1169 plus deletion of nt 426 (HDV-1 d1); and one harbored the same mutation at nt 1169 plus deletion of nt 426-428 (HDV-1 d3). These four replication-competent HDV-1 mutants (Supplementary Figure 4) were co-transfected or separately transfected with a WT HDV-4 sequence, and RT-PCR reactions using clade-specific primer pairs were performed. Again, fifteen cloned HDV plasmids for each HDV recombinant were subjected to SacII/BstBI-RFLP assays. As summarized in Table 1, the most frequently occurring crossover site on the 5’-(1-4)-3’ recombinants was shifted to a region covering nt 1071-1155 for all three of the new mutants. Sequencing indicated that 88.9-90% of these recombination events occurred at nt 1132-1146. Our finding that abrogation of the nt 424-428/1164-bulge shifted the bulk of the crossovers to the previously second-most frequent crossover site (nt 1132-1146, as observed in Figure 1C when consensus primers were used in RT-PCR) further confirmed the importance of this intrinsic asymmetric bulge in promoting template switching at nt 1157-1205. In contrast, the SacII-BstBI-RFLP results obtained from the 5’-(4-1)-3’ recombinants for all of the mutants were similar to those observed for the HDV-1 M1 mutant. Since the bulge on the HDV-4 genome remained intact, it is reasonable that the 5’-(4-1)-3’recombination patterns would not be affected if recombination occurred predominantly during the synthesis of HDV genomic RNA.

**Table 1:** Effect of the bulge-reducing mutants on distribution of the recombination junctions occurring at nt 1071-1269.

Recombination patterns	Recombination frequency (%)				
	**Crossover region (nt)**	**HDV-1 mutant used in co-transfections^**			
		**M1**	**M2**	**d1**	**d3**
5′-(1-4)-3′ 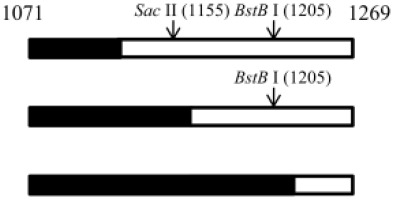	1071-1155	20.0	66.7 (90.0)^#^	60.0 (88.9)^#^	53.3 (88.9)^#^
		20.0	66.7 (90.0)^#^	60.0 (88.9)^#^	60.0 (88.9)^#^
	1155-1205	66.7 (90.0)*	20.0	20.0	26.7
		66.7 (93.8)*	20.0	26.7	13.3
	1205-1269	13.3	13.3	20.0	20.0
		13.3	13.3	13.3	26.7
5′-(4-1)-3′ 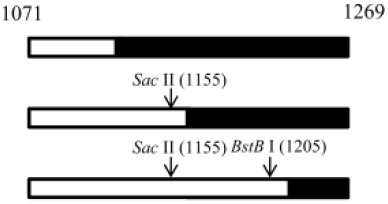	1071-1155	20.0	20.0	26.7	20.0
		13.3	13.3	-	-
	1155-1205	53.3	53.3	60.0	66.7
		60.0	60.0	-	-
	1205-1269	26.7	26.7	13.3	13.3
		26.7	26.7	-	-

^^^: SacII-BstBI-RFLP data are shown. The most frequent crossovers are shadowed.

*^,#^: The percentages of crossovers occurring at nt 1157-1168 and nt 1132-1146, respectively, as assessed by sequencing of the cloned PCR products, are given in parentheses.

## DISCUSSION

Homologous recombination is a universal genetic process that plays multiple roles in the biology of all organisms, including viruses. In DNA viruses, recombination occurs via a break-and-rejoin mechanism similar to that seen in eukaryotes and bacteria [41]. Although different RNA viruses can vary in their genomic structures and replication strategies, they use similar mechanisms to undergo RNA recombination, which contributes to maintaining their variability and infectivity. The replicating template-switching mechanism appears to be the most common mechanism for homologous RNA recombination in retroviruses as well as in many RNA viruses that encode viral RdRps [21, 42]. Interestingly, HDV is the only human pathogen that uses the host RNAP, which is normally a DNA-directed RNA polymerase, for viral RNA-dependent RNA replication [1, 2]. HDV can also undergo traditional homologous RNA recombination between two replicating HDV RNAs. To date, however, we have not fully elucidated the molecular mechanisms underlying HDV RNA recombination. Our previous and present data on RNA recombination between two replicating HDV RNAs in natural infections and cultured cells indicate that: (i) HDV RNA recombination is faithful and homologous; (ii) recombination occurs predominantly during the synthesis of genomic HDV RNA; and (iii) viral RNA structure promotes template-switching for HDV genetic recombination. These data expand our understanding of the distinct template-switching capacities of mammalian RNAPs on replication-competent HDV RNA genomes. Furthermore, HDV resembles the plant pathogens known as viroids, which are a group of small non-coding RNAs, in terms of its genomic structure and replication strategy. Interestingly, various reports have claimed to show RNA recombination between plant viroids [43, 44]. Therefore, RNA recombination represents another feature that is shared between HDV and viroids.

**Figure 6:**
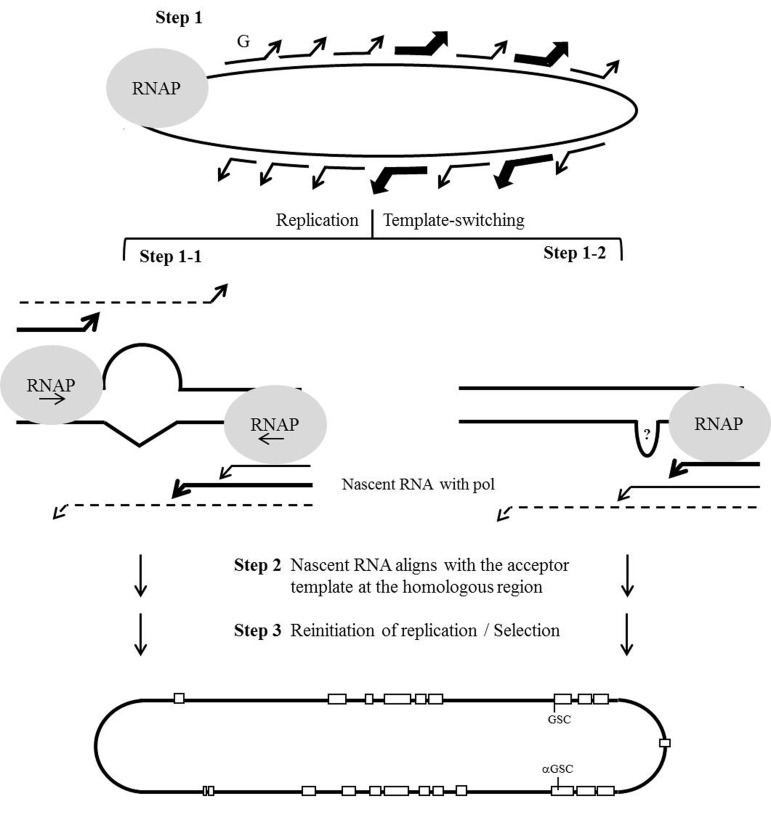
Schematic description of the hypothesized template-switching mechanism for HDV RNA recombination The gray circle symbolizes the replication complex containing the host RNAP. Lines with arrowheads represent the newly synthesized nascent-strand RNAs that, along with the replication complex, are undergoing template-switching randomly. Bold lines with arrowheads indicate the template-switching promoted by HDV RNA structures (step 1). Dashed lines with arrowheads represent nascent RNAs whose 3’-ends are imperfectly aligned with their acceptor templates (steps 1-1 and 1-2). Recombination sites that are homologous between the HDV-1 and HDV-4 RNAs are depicted as open squares. The individual steps in the template switching of HDV RNAs by host RNAP are described in the text.

Based on the data presented in the present study, a model for host RNAP-driven and viral RNA structure-assisted HDV RNA recombination is proposed. As shown in Figure 6, we hypothesized that the nonprocessivity of the host RNAP on an atypical viral RNA template leads to template-switching occurring frequently and randomly across the whole genome (step 1). In some cases, RNA secondary structures (such as an intrinsic bulge shown in Figure 5) may promote template switching at two homologous regions located on opposite sides of the HDV rod-like genome (step 1-1). Some yet-unidentified RNA structures could also serve as a signal that causes the polymerase to pause (step 1-2). The nascent RNAs and the associated transcription factors dissociate from the donor template and subsequently align with the acceptor template precisely at the homologous recombination site, where it undergoes faithful RNA recombination (step 2) [27, 39]. After re-initiation of replication on the acceptor template, recombined genomes with survival advantages are selected (step 3). RNA structures and selection pressures would thus result in the nonrandom distribution of recombination. Although HDV does not encode an RdRp, it undergoes homologous HDV RNA recombination via a host RNAP-mediated template-switching mechanism involving viral RNA structures. Therefore, our proposed template-switching recombination model for HDV is similar to that proposed for other animal viruses, except that our model involves host RNAP performing template-switching and viral ribozyme activity generating a crossover. HDV RNA recombination is thereby an interesting issue not only in virology, but also in the molecular biology of transcription. Notably, mammalian RNAP, which is normally a DNA-directed RNA polymerase, is involved in HDV RNA-directed RNA transcription, mutation, template-switching, and recombination. Moreover, the three major mechanisms through which genetic variations are known to enter the HDV genome (polymerase incorporation errors, host ADAR1-catalyzed amber/W editing, and host RNAP-directed RNA recombination) all rely on the activities of host enzymes.

We might then ask what other yet-unidentified RNA structures might serve as host RNAP-pausing signals. HDV RNA replication occurs via a rolling-circle mechanism that is highly dependent on viral RNA structures, specifically the unbranched rod-like RNA structure and the alternative pseudoknot ribozyme structure [45]. The HDV-1/HDV-4 recombination map presented herein indicates that nt 685-710, nt 723-745, and nt 747-772 in the genomic pseudoknot ribozyme domain, as well as nt 812-839, nt 844-874, and nt 876-911 in the antigenomic pseudoknot ribozyme domain (Figure 1C) serve as recombination “hot spots”. Moreover, our localization of two inter-clade junctions of the two identified recombinant clones to nt 685-710/747-772 and nt 812-839/876-911, respectively, further supports the hypothesis that pseudoknot structure, its intermediates, or incorrectly folded ribozyme structure, efficiently promote progressive pausing and template-switching by the host RNAP. These data support the critical notion that functionally important RNA structures contribute to the evolution of HDV.

The finding that the nt 424-428/1164-bulge promotes HDV RNA recombination prompted us to search for similar RNA structure across the whole genome. We identified only a single relevant bulge in the entire the HDV rod-like RNA genome. Located at nt 126/1463-1467, it is identical in size and structure to the recombination-promoting nt 424-428/1164-bulge. However, this so-called nt 126/1463-1467-bulge is located within a region that is categorized as a recombination “cold spot” (Figure 1C). Further analysis revealed that nt 126 is located in a region that is highly heterogeneous between the HDV-1 and HDV-4 sequences, and thus does not favor homologous RNA recombination. If recombination occurs near nt 1463-1467 but not at nt 126, the unbranched rod-like structure of the HDV genome will not be maintained. Furthermore, recombination occurring near nt 1463-1467 will lead to the production of a chimeric HDAg with a junction that maps to the N-terminal region of HDAg, which has been shown to be insufficient to trans-activate HDV RNA replication (Figure 2C). These observations are consistent with the model proposed in Figure 6, in which several factors contribute to the generation of “hot spots” for HDV RNA recombination, including polymerase pausing signals for template-switching, sequence homology between two parental sequences, and biological selection of the resulting recombinants. The HDV RNA folds into an unbranched rod-like structure that comprises segments with 11 consecutive base pairs (using Italian HDV-1 sequence as an example) and is interspersed with small internal bulges and loops. Since bulges are distributed throughout the genome, future studies should investigate whether and how bulges having different sizes/structures can affect HDV RNA recombination.

After template-switching, the nascent RNA must anneal with the acceptor template to re-initiate replication. In HDV, homologous recombination junctions can be as short as 5 nt (e.g., nt 1513-1517 in the map shown in Figure 1C; nt 415-419, 982-986, and 1561-1565 in the HDV-1 intra-clade recombination map reported recently and summarized in Supplementary Figure 5) [28]. However, most of the crossovers identified in the HDV inter- and intra-clade recombination maps were more than 10 nt in length. Previous studies showed that very short stretches (2 to 5 nt) of sequence complementarity were sometimes sufficient to promote re-initiation of the RdRp among plants viruses *in vitro* [46, 47]. The mammalian RNAP, however, seems to require longer complementary sequences to re-initiate RNA replication on its atypical HDV RNA templates. It has also been reported that AU-rich stretches promote recombination in the donor RNA of brome mosaic virus and human immunodeficiency virus [48, 49, 50]. The HDV genome is GC-rich, and we did not identify any AU-rich region at or adjacent to the crossovers in our HDV intra- and inter-clade recombination maps (Supplementary Figure 1; [28]).

We compared the recently published HDV-1 intra-clade recombination map [28] with the HDV-1/HDV-4 inter-clade recombination map established in the present work (Supplementary Figure 5). Fifty homologous recombinants (60 recombination events) and 54 recombinants (57 recombination events) were obtained by analyzing 200 and 400 cloned PCR products obtained from cells co-transfected with two HDV-1 clones (89% sequence homology) and with HDV-1 plus HDV-4 (78% sequence homology), respectively. These results were consistent with the published data indicating that a higher sequence homology between two HDV sequences was associated with a higher recombination frequency [27]. A total of 22 crossovers were identified in the intra- and inter-clade recombination maps. As summarized in Supplementary Figure 5, 18 of the crossovers identified in the HDV-1/HDV-4 inter-clade recombination map were also demonstrated in the HDV-1 intra-clade recombination map. More than 90% sequence homology between HDV-1 and HDV-4 was observed in the three most frequently occurring recombination regions, which were located at nt 295-486, nt 685-911, and nt 1069-1355. HDV-1/HDV-4 inter-clade RNA recombination was seldom observed in two relatively low homology (<70%) domains of the HDV RNA. The first one was located at one end of HDV rod-like RNA genome (nt 1360-290) (Figure 1C). It included the potential RNA promoter for viral RNA synthesis (nt 1541-60) and the region encoding the N-terminal 80 aa of HDAg. The second domain was located at nt 487-684/912-1068 and corresponded to the C-terminal 19-aa extension of L-HDAg (Figure 1C) and its opposite side on the HDV rod-like RNA genome. In contrast, the region encoding the C-terminal 19 aa region, which was highly conserved in strains of the same HDV clade (96%), was detected in the HDV-1 intra-clade recombination map (Supplementary Figure 5). The overlap of these functionally important domains with the most divergent regions among the HDV clades ensures that these molecular signatures are not interrupted by RNA recombination during HDV evolution.

We have previously studied HDV RNA recombination by using clade-specific RT-PCR primer pairs to specifically amplify the 5’-(1-4)-3’ and 5’-(4-1)-3’ recombinants (covering nt 940 to 1271) (fragment A’) (Supplementary Figure 5; [20]). Here, we employed a similar strategy to amplify a region covering nt 243-700 (fragment C’) (Supplementary Table 1 and Supplementary Figure 5). As described previously, this method had the benefit that the clade-specific primers were able to suppress potential artificial recombination during PCR [51]. To investigate the recombination junctions, PCR products were cloned into T vectors, and 10 clones each of the 5’-(1-4)-3’ and 5’-(4-1)-3’ recombinants were subjected to sequencing analysis. As summarized in Supplementary Figure 5, the crossover regions were mapped to nt 295-329, 391-435, 443-458, and 465-486. Furthermore, three pairs of crossovers identified in fragments A’ and C’ (those at nt 391-435/1157-1205, 443-458/1132-1146, and 465-486/1108-1123) were each located exactly opposite one another on the HDV rod-like structure. All of crossovers identified in fragment C’ were also detected in recombinants when the consensus primers were used during PCR (Figure 1C and Supplementary Figure 5). Detection of the same recombination junctions by two independent analyses further proved that the observed HDV RNA recombination occurred in the co-transfected cultured cells rather than being a random event.

Studies regarding the replication-competence of HDV recombinant genomes have indicated that an intact rod-like structure is strictly required for HDV RNA replication. These data are also consistent with the notion that the structure of the HDV genome makes a major contribution to redirecting the host RNAP to recognize the circular HDV RNA as a template. As expected, HDV fails to replicate when the rod-like structure of the middle region of recombinant R2 is interrupted. Surprisingly, R2 expresses HDAg to 15% of the WT level (Figure 3C, lane 4). A previous report indicated that only a small amount of HDAg (less than 5% of that expressed from a WT replication clone) is made by HDV RNAs transcribed from an endogenous promoter on the HDV cDNA [40]. It therefore remains possible that a partial rod-like RNA structure formed locally at one end of the HDV genome (i.e., that containing the potential HDV RNA promoter) is sufficient to drive transcription of the HDAg mRNA. The molecular mechanisms responsible for the regulation of HDV genome replication and HDAg mRNA transcription are an interesting question remains to be elucidated.

Functional differences among HDV clades have been reported. For example, clade-specific complementation of HDV RNA replication by S-HDAg has been reported between HDV-1 and HDV-3 [31]. In contrast, although the HDAg of HDV-1 failed to support the replication of HDV-2 RNAs, the HDAg of HDV-2 was found to trans-activate HDV-1 RNA synthesis [52]. Here, we found that the HDV-1 and HDV-4 HDAgs both failed to support RNA replication of the other clade. HDV-4 was originally named “genotype IIb” because it formed a monophyletic group that was most closely related to HDV-2 [53, 54]. However, a subsequent phylogenetic study suggested that HDV-4 was distinct from HDV-2 [55]. Our observation that the S-HDAg of HDV-4 failed to trans-activate HDV-1 RNA replication further indicated that they differed not only in their sequences, but also in their biological functions.

As shown in Figure 4D, of the 70 picked colonies, three clones were found to represent a recombinant pattern that the inter-clade junction of HDV recombinants could be generated by self-cleavage/ligation. Interestingly, 32 clones could be amplified by primer pairs a and c but not by primer pair b. These recombinants were generated by a second recombination event occurs upstream of the self-cleavage site. The remaining 35 clones could only be amplified with primer pair b, which were produced by two recombination events, one in the homologous region immediately downstream the self-cleavage site and another opposite this region. Our data were thereby consistent with the notion that most of the HDV recombinants identified here carried even number of real recombination junctions.

Similar experimental design as employed in Table 1 was used to detect the effect of the same nt 424-428/1164-bulge bulge on recombination occurring at a region that was opposite nt 1157-1205 on the HDV rod-like genome (Supplementary Table 2). RT-PCR reactions using clade-specific primer pairs (nt 243-700), were performed using RNA extracted from cells co-transfected with vectors encoding various HDV-1 mutants (M1, M2, d1, or d3) and WT HDV-4. Cloned PCR plasmids were then subjected to the BstX1/BstBI-RFLP assays. The cutting sites for BstX1 (nt 358) and BstBI (nt 528) are present in HDV-4 and HDV-1, respectively (Supplementary Table 2, left column). As summarized in Supplementary Table 2, the most frequently occurring crossover site on the 5’-(1-4)-3’ recombinants was shifted from a region spanning nt 358-528 for M1 mutant to a region covering nt 243-358 for the M2, d1, and d3 mutants. In contrast, RFLP results obtained from the 5’-(4-1)-3’ recombinants for all of the bulge-reducing mutants were similar to that observed for the HDV-1 M1 mutant (nt 358-528). Taken together, these data strongly suggest that HDV RNA recombination occurred predominantly during the synthesis of HDV genomic RNA and that the asymmetric nt 424-428/1164-bulge on the HDV rod-like RNA promoted HDV RNA recombination events occurring on the opposite sides of the HDV rod-like RNA genome.

Genetic diversity contributes to the phenotypic diversity of HDV. In a recently published paper and the present work, we demonstrate that intra-clade chimeric S-HDAg harboring crossovers identified in cultured cells co-transfected with two HDV sequences can support viral replication, even to a level higher than that of WT S-HDAg [28]. In the present study, the Sm-1^101/116^4 S-HDAg chimera supported replication of HDV-1 at 180% of the level seen with WT HDV-1 HDAg (Figure 2C). A previous study showed that the C-terminal aa 147-195 of the HDV-1 and HDV-3 S-HDAgs each increased replication when grafted onto the other HDAg [31]. The HDV RNA genome has evolved together with its specific HDAg gene. The observation that the trans-complementation activity was stronger for chimeric HDAg than WT HDAg suggests that some yet-unidentified functional element(s) at the C-terminus of S-HDAg might play an important role in limiting viral replication, thereby favoring the establishment of chronic HDV infection. In the future, detailed studies regarding the trans-activation abilities of HDAg chimeras should improve our understanding of the molecular mechanisms underlying HDV replication. Moreover, the successful detection of recombinants with functional HDAg and correct genomic structures in cultured cells (Figures 2 and 3) suggests that such recombinants have the potential to survive in nature. Indeed, phylogenetic and recombination studies have identified potential naturally occurring HDV-1/HDV-2 and HDV-4/HDV-4M recombinants in Vietnam and Miyako Island, Japan, respectively [27, 29]. However, we do not have direct evidence demonstrating that certain recombinants observed in co-transfected cultured cells would be selected over passages in cell culture and/or in animals. Future experiments should include co-transfection of HDV and HBV followed by characterization of the released HDV genomes, and the use of released HDV recombinants to infect experimental animals or cultured cells expressing viral receptor sodium taurocholate co-transporting polypeptide [56].

The prevalence of HDV infection has significantly declined in developed countries, mainly due to the implementation of HBV vaccination programs [57, 58]. However, HDV remains a medical issue in the developing world, where HBV remains unchecked. The number of HDV patients stopped decreasing towards the end of the 1990s in Europe, mainly because of increased immigration from endemic regions [59, 60]. Multiple-clade infections can occur in patients at high risk of repeated exposure [61]. It has been suggested that genetic recombination can occur both between and within HDV clades [19, 20, 28]. Thus, we should be aware of the generation of naturally occurring HDV recombinants especially in regions circulating multiple HDV clades. Such recombination events could contribute to the emergence of new strains with different degrees of replication and pathogenicity.

## MATERIALS AND METHODS

### Establishment of the HDV-1/HDV-4 recombination map

To initiate HDV genome replication, we referred to the previous reports [20, 34]. Briefly, we co-transfected COS7-SmT1 cells stably expressing S-HDAg with equal amounts of *in vitro*-transcribed WT HDV-1 and HDV-4 genomic RNA monomers, using the SuperFect transfection reagent (Qiagen). At 6 days post-transfection, total cellular RNA was extracted using TriReagent (Invitrogen) and subjected to RQ DNase I (Promega) treatment. To establish the recombination map, RT-PCR amplification was performed using the Titan One Tube RT-PCR System (Roche), as described in a previous report [20]. The consensus primer pairs used for fragments A∼D were 18/55’ (nt 886-1308; [20]), 88/87, 86/74, and 80/81, respectively (summarized in Supplementary Table 1). The nucleotide (nt) numbering system used here is in accordance with that of Kuo et al. (1988) [7]. RFLP analyses were used to differentiate the HDV-related sequences present in the co-transfected cells. The restriction enzymes (RE) used in the RFLP assays and the predicted RFLP profiles (including only the sizes of the larger bands observed after digestion) are summarized in Figure 1A. The products obtained from the RT-PCR-RFLP assays were separated on a 3% agarose gel and stained with ethidium bromide. To rule out the possibility that the detected bands were artifacts generated during RT-PCR, we routinely performed RT-PCR-RFLP assays of mixed parental RNAs obtained from separately transfected cells. Worth mentioning, the presence of potential recombinants in co-transfected cells but not in the control mixed samples was taken as an initial and essential criterion for the subsequent analyses of HDV recombination in the co-transfection experiments, as shown in Figure 1B and Supplementary Figure 6 and as described in previous reports [20, 27, 28]. The purified PCR products were also cloned into T-vectors (TOPO TA Cloning Vector; Invitrogen). Plasmids were extracted from 100 colonies for each PCR product, digested with BstXI (for fragment B) or EcoRI (for fragments C and D) to release the HDV cDNA inserts, and then further digested with the enzymes indicated in Figure 1A to differentiate the HDV clades and recombinants. For fragment B, SalI-BglII-RFLP assay was initially used to identify potential recombinants with crossovers located between SalI and BglII sites (nt 1353-224). The published XhoI-RFLP assay for amplified fragment A (nt 886-1308; HDV-1 and HDV-4 each have a single XhoI site at nt 1272 and 969, respectively) [20] identified potential recombinants with crossovers located between nt 969-1272. Therefore, another RFLP assay for fragment B should be performed to detect the potential recombinants with crossovers occurring between XhoI (nt 1272) and SalI (nt 1353) sites. Our sequence analysis indicated that HDV-1 had three XhoI sites (at nt 1272, 1506, and 1545), whereas HDV-4 had only one (nt 1506) (Figures 1A). A second RFLP assay for fragment B, performed by double-digestion with BstXI (to release the HDV cDNA inserts) and XhoI, was then used to identify potential recombinants with junctions located in the range of nt 1272-1353. The two largest XhoI-cleaved bands for parental HDV-1 and HDV-4 would be 458/234 bp and 497/295 bp, respectively. If one recombination event occurred between the XhoI sites located at nt 1272 and 1506, the two largest XhoI-cleaved bands for the 5’-(1-4)-3’ and 5’-(4-1)-3’ recombinants would be 497/234 bp and 458/295 bp, respectively. Twenty clones of each HDV-1 and HDV-4 candidate and all of the clones with recombinant RFLP patterns were subjected to sequence analysis using an ABI377 DNA sequencing system (Perkin-Elmer/Applied Biosystems).

### Plasmids for HDV genome expression

pSVL-D_2_I and pSVL-D_2_IIb were replicating HDV constructs that expressed the genomic RNAs of HDV-1 and HDV-4, respectively [16, 33]. pSVL-D2M (herein called pSVL-D_2_1M) expressed an HDV-1 RNA having a two base-pair deletion in the HDAg ORF [16]. Similarly, pSVL-D_2_4M, which contained an HDV-4 head-to-tail NheI-NheI(431) mutated dimer, expressed an HDV-4 RNA harboring a deletion in the HDAg ORF.

As mentioned above, TOPO plasmids containing parental or recombinant HDV inserts were obtained from RT-PCR products amplifying from co-transfected cells. Four plasmids containing cloned PCR products were used to construct HDV recombinant genomes R1 and R2 which contain recombination junctions mapping to nt 391-435/1157-1205 and nt 747-772/1157-1205, respectively. They are TOPO-AR94, -BI31, -CR93, and -DR58 (here, the letters A, B, C, and D represent the corresponding RT-PCR fragments, while R and I represent recombinant and parental HDV-1 clones, respectively). TOPO-AR94 contained the 5’-(4-1)-3’ recombinant sequence with the junction mapping to nt 1157-1205; TOPO-CR93 and TOPO-DR58 contained 5’-(1-4)-3’ recombinant sequences with crossovers located at nt 391-435 and 747-772, respectively; and TOPO-BI31 contained a parental HDV-1 sequence. pGEM-T-1.1xIIb contained a 1.1-mer (nt 260-449) of the HDV-4 cDNA [33]. The recombinant genomes were constructed using overlapping extension (OE)-PCR [62] and a standard molecular cloning protocol involving multiple RE digestion and ligation reactions [63], as summarized in Supplementary Figure 7. Finally, the unit-length 1.7-kb NheI-NheI(431) fragments were excised from pGEM-T-1.1-R1 and pGEM-T-1.1-R2 and inserted into the XbaI site of the pSVL vector. The plasmids containing genomically oriented head-to-tail dimers of the 1.7-kb NheI-NheI(431) fragments of the HDV recombinant sequences were obtained and designated pSVL-D_2_R1 and pSVL-D_2_R2, respectively.

### Plasmids for expression of S-HDAg

Six plasmids encoding HDV-1/HDV-4 chimeric S-HDAgs were constructed. The S-HDAg ORFs contained in pSVL-D_2_I, pSVL-D_2_IIb, and pSVL-D_2_R1 were PCR amplified with primers 77/78 (Supplementary Tables 1 and 3), and the gel-purified products were sub-cloned into the PCR3.1 T-vector (Promega). Clones were subjected to RE digestion and sequencing to confirm that they contained the correct sequences in the proper orientation relative to the human cytomegalovirus immediate-early promoter. The resulting plasmids were designated pCR3.1-Sm1, pCR3.1-Sm4, and pCR3.1-Sm1^125/147^4. The first two encoded the WT small HDAgs of HDV-1 and HDV-4, respectively, while the third expressed a chimeric HDAg in which the N-terminal 124 aa corresponded to the HDV-1 sequence; aa 125-147 comprised a region conserved between HDV-1 and HDV-4; and the remaining sequence came from HDV-4. To construct pCR3.1-Sm4^125/147^1, we PCR amplified two overlapping HDV cDNA fragments (corresponding to positions 886-1308 and 1211-1631) from TOPO-AR67 [whose insert contained the 5’-(1-4)-3’ recombinant sequence with the junction mapped to nt 1157-1205] and pSVL-D_2_IIb using primer pairs 18/55’ and 88/78, respectively. The overlapping PCR fragments were joined by OE-PCR using primer pair 77/78. The resulting PCR product was gel-purified and cloned into pCR3.1. The generated plasmid was subjected to sequencing confirmation of the correct sequence and proper orientation, and designated pCR3.1- Sm4^125/147^1. Similar OE-PCR strategy was also used to generate chimeric HDAgs Sm-1^101/116^4 and Sm-4^101/116^1, which had a crossover mapped to nt 1255-1274, and control Sm-1^48/54^4 and Sm-4^48/54^1, which had a junction that was not identified in the inter-clade recombination map. The utilized templates and primer pairs are summarized in Supplementary Table 3. The primer sequences are presented in Supplementary Table 1.

### Examination of HDV genomes and proteins

COS7 monkey kidney cells were plated in six-well (35-mm-diameter) dishes and transfected with 4 μg of DNA per well, using the Lipofectamine reagent (Invitrogen), as described elsewhere [28]. At 6 days post-transfection, HDV RNA and HDAg were analyzed as markers for HDV replication using NB and WB analyses, respectively, as described previously [33]. *In vitro*-transcribed monomeric HDV-1 RNAs were used as the hybridization probes for NB. A previous study showed that the HDV-4 RNA could be identified with an HDV-1 RNA probe under the utilized hybridization and washing conditions [33]. All experiments were repeated at least twice.

### Detection of a genomic self-cleavage site that serves as an inter-clade junction

The expression vector, pECE-GA, which was kindly provided by Dr. Michael Lai (Academia Sinica, Taiwan), consists of a dimer of HDV-1 cDNA carrying a mutation 3’ of the genomic self-cleavage site [40]. The PstI-PstI (nt 659) HDV cDNA monomer was excised from pECE-GA and subcloned into pGEM-T (Promega) to yield pGEM-T-GA, which was used for *in vitro* transcription. COS7-SmT1 cells were co-transfected with *in vitro*-transcribed HDV-1 GA and HDV-4 genomic RNA [20], and the role of self-cleavage reaction in HDV RNA recombination was assessed. The relative amounts of the two HDV RNAs were adjusted to obtain similar band intensities for parental sequences in an XhoI-RFLP assay of RT-PCR products amplified with consensus primers 18/55’ [34, 53]. These RNA samples were then subjected to nested PCR using a HDV-4 primer pair 471-4f/1045-4r (nt 471-1045) for the first round of amplification and a recombinant primer pair 574-4f/730-1r (nt 574-730) for the second (nested) PCR (Supplementary Table 1). The resulting PCR products were cloned into a T-vector. To distinguish the molecular natures of the HDV recombinants, 70 colonies were subjected to colony-PCR reactions using primer pairs a, b, and c [64]. The forward primer was 574-4f in all three cases, whereas the reverse primers were 686A, 686G, and 683A, for primer pairs a, b, and c, respectively (Supplementary Table 1).

### Investigation of HDV RNA recombination-promoting RNA structure

A C-to-T base substitution at nt 1169, which disrupted the recombination junction spanning nt 1157-1205 into two smaller domains, was introduced into the HDV-1 cDNA using a single-step, single-primer PCR-based mutagenic technique [65]. pG4Z-D1I, which contained 1.7-kb XbaI-XbaI(781) cDNA of HDV-1 [33], served as the PCR template and P-M1 was used as the primer (Supplementary Table 1). The resulting mutant was designated pG4Z-D1M1. A similar mutagenesis strategy was employed to generate the deletion mutants that lacked the proper nt 424-428/1164-bulge. The RNA structure was predicted using RNAstructure Version 3.7 [66]. Using pG4Z-D1M1 as the PCR template and P-d1 and P-d3 as the primers (Supplementary Table 1), we generated pG4Z-D1d1 and pG4Z-D1d3, respectively. Two sequential PCR reactions were performed to generate the M2 mutant. The first PCR used pG4Z-D1M1 as the PCR template and P-M2-1 as the primer. The resulting plasmid, pG4Z-D1M2’, was used as the template for a second PCR with primer P-M2-2 to generate pG4Z-D1M2 (Supplementary Table 1). All of the obtained mutants were confirmed by sequencing. The 1.7-kb XbaI-XbaI(781) fragments of the HDV-1 mutants were excised from the pG4Z constructs and cloned into XbaI- and CIP-pretreated pCR3.1 (Promega). Plasmids containing head-to-tail dimers of the HDV-1 mutants in a genomic orientation were obtained and designated pCR3.1-D_2_M1, -D_2_d1, -D_2_d3, and -D_2_M2, as appropriate. Similarly, pCR3.1-D_2_IIb was replicating HDV constructs that expressed the genomic dimer RNA of WT HDV-4. Our results revealed that all of the mutants were replication-competent, albeit with different replication abilities (Supplementary Figure 4). These HDV-1 mutants were then co-transfected into COS7 cells along with pCR3.1-D_2_IIb (expressing HDV-4 genome), and RNA samples were harvested at 6 days post-transfection. A constant amount of the HDV-4 genome expression plasmid was co-transfected with varied amounts of the HDV-1 mutants to achieve similar band intensities between the parental sequences, as judged by RT-PCR-XhoI-RFLP of the region amplified by primers 18/55’ (nt 886-1308) [34]. pCR3.1 DNA was added to ensure a constant amount of total DNA per co-transfection. All experiments were performed at least twice, except when otherwise noted. A region covering nt 1071-1269 was RT-PCR amplified using clade-specific primer pairs F-1-1/R-4-1 and F-4-1/R-1-1 (Supplementary Table 1) to assess 5’-(1-4)-3’ and 5’-(4-1)-3’ recombinants, respectively. Cloned PCR products [15 clones each for the 5’-(1-4)-3’ and 5’-(4-1)-3’ recombinants] were subjected to SacII/BstBI-RFLP assays. Similarly, another region spanning nt 243-700 was RT-PCR amplified using clade-specific primer pairs 5’-1/3’-4 and 5’-4/3’-1 (Supplementary Table 1) to assess 5’-(1-4)-3’ and 5’-(4-1)-3’ recombinants, respectively. Cloned PCR products [15 clones each for the 5’-(1-4)-3’ and 5’-(4-1)-3’ recombinants] were subjected to BstXI/BstBI-RFLP assays.

## SUPPLEMENTARY MATERIALS FIGURES AND TABLES





## Abbreviations

HDV: Hepatitis delta virus
RNAP: RNA polymerase
RdRp: RNA-dependent RNA polymerase
HDAg: Hepatitis delta antigen
ADAR1: Adenosine deaminase acting on RNA 1
RFLP: Restriction fragment length polymorphism
RT-PCR: Reverse transcription-polymerase chain reaction
NB: Northern blotting
WB: Western blotting
OE-PCR: Overlapping extension-PCR
WT: Wild-type
ARM: Arginine-rich motif
RBD: RNA binding domain
CC: Coiled-coil domain
NLS: Nuclear localization signal
