# Supplementary material for: Analyses of a whole-genome inter-clade recombination map of hepatitis delta virus suggest a host polymerase-driven and viral RNA structure-promoted template-switching mechanism for viral RNA recombination
Source: Oncotarget. 2017 Jun 1;8(37):60841–59. doi: 10.18632/oncotarget.18339 (PMC5617389; doi:10.18632/oncotarget.18339)
Supplement: Supplementary file 1 [file oncotarget-08-60841-s001.pdf]

Analyses of a whole-genome inter-clade recombination map of hepatitis delta virus suggest a host polymerase-driven and viral RNA structure-promoted template-switching mechanism for viral RNA recombination

SUPPLEMENTARY MATERIALS

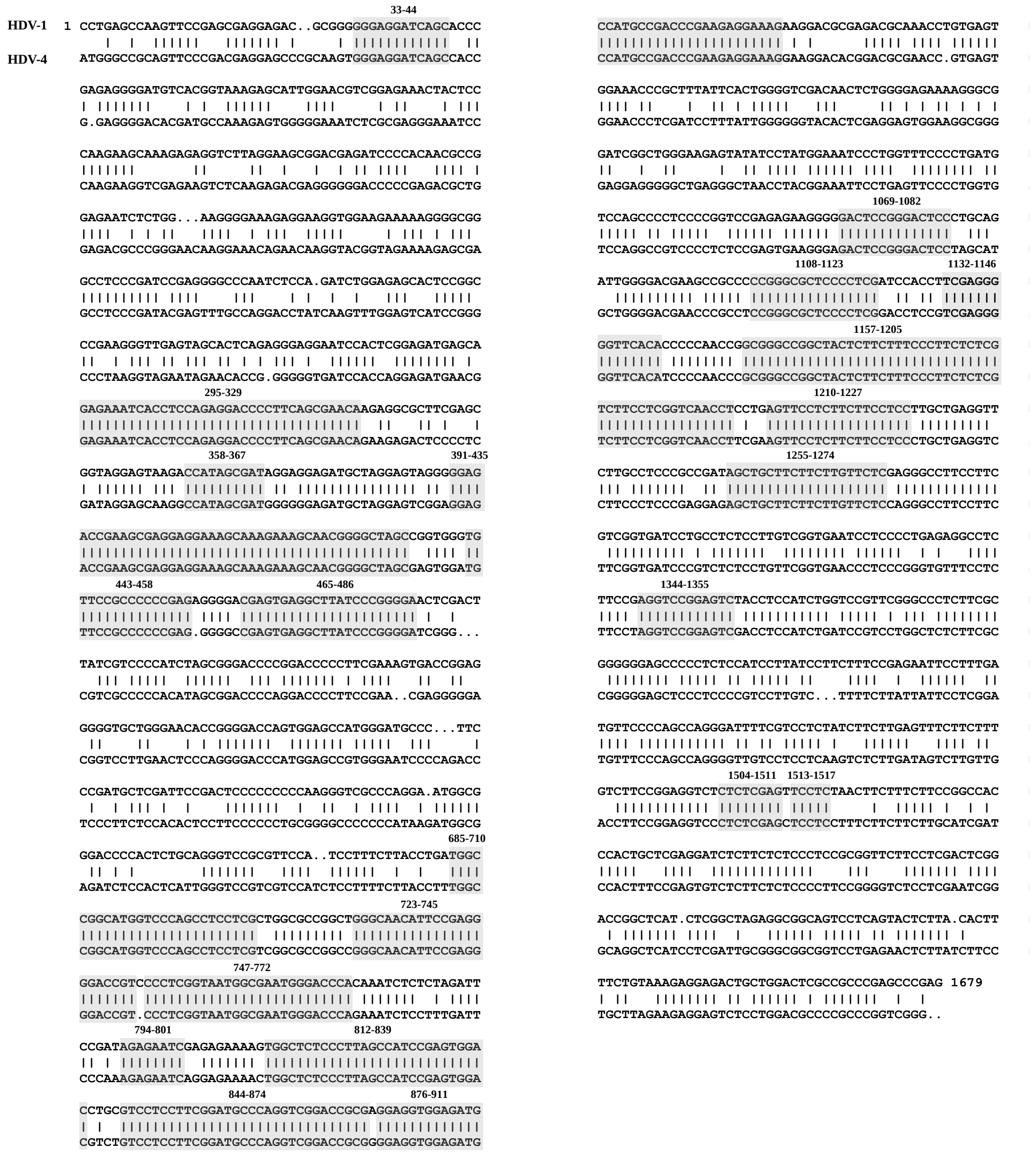

Supplementary Figure 1. Primary structures of the crossover regions for the inter-clade HDV-1/HDV-4 recombinants. Genomic sequences are given. Short lines indicate bases that are homologous between the HDV-1 and HDV-4 sequences. The crossover regions are shaded gray.

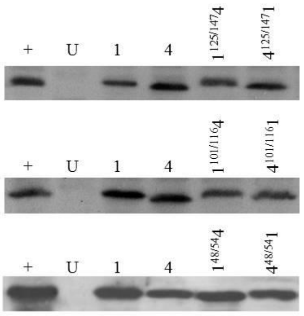

Supplementary Figure 2. Expression of various HDV-1/HDV-4 HDAg chimeras. HDAg expression was determined by Western blotting analyses, respectively. Lanes: +, protein extracted from a cell line stably expressing HDV-1 S-HDAg; U, untransfected cells; 1, HDV-1 HDAg; 4, HDV-4 HDAg.

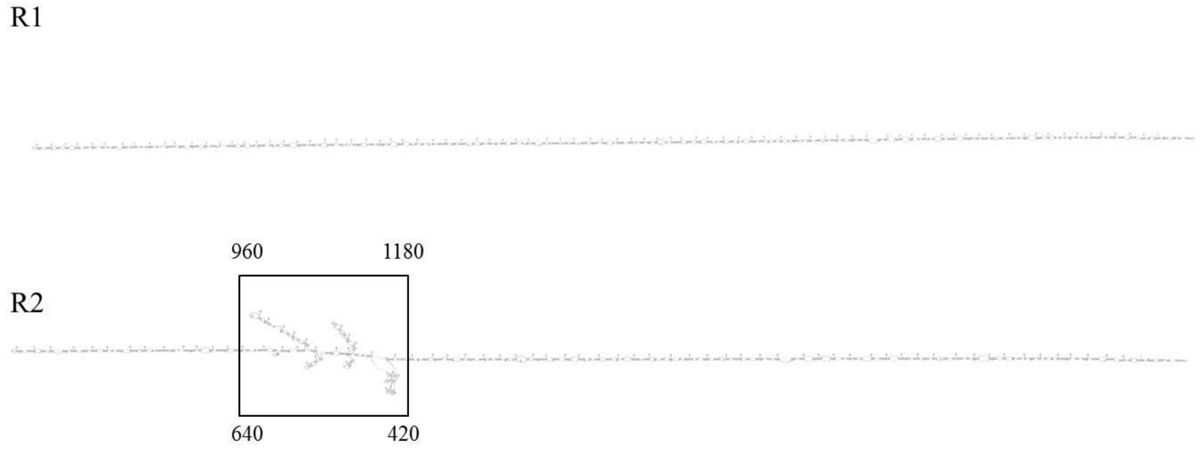

Supplementary Figure 3. The HDV RNA structure of recombinant R1 and R2. The unbranched rod-like structure of R1 genome, but not R2 genome, is maintained. The branched region on R2 genome is boxed.

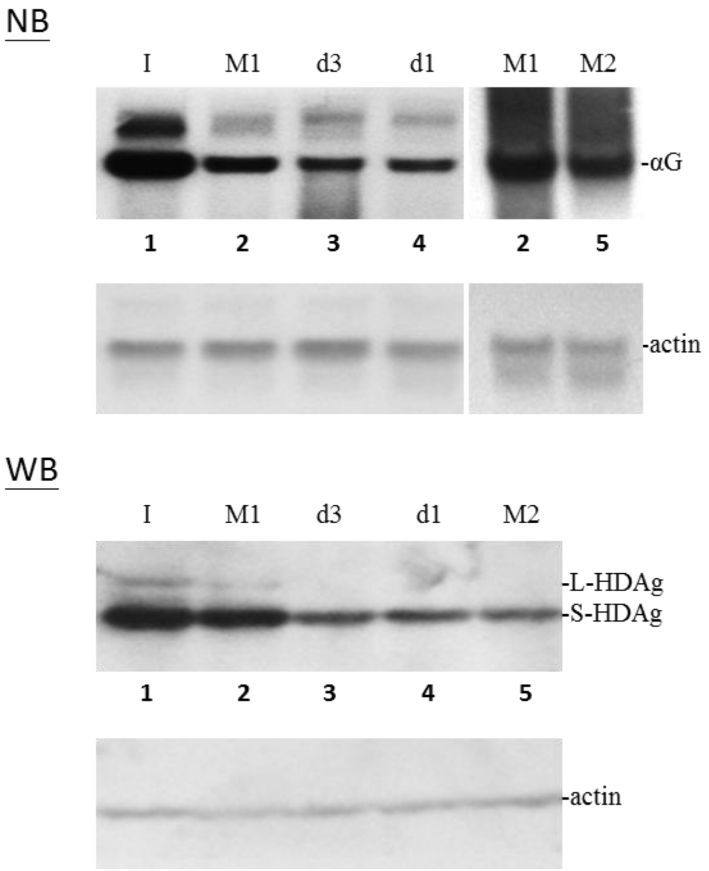

Supplementary Figure 4. HDV replication and HDAg expression of various HDV mutants. The HDV replication and HDAg expression levels of various HDV mutants were determined by Northern blotting (NB) and Western blotting (WB) analyses, respectively. Lanes: 1~5, samples extracted from cells transfected with plasmids expressing RNAs for WT HDV-1 and HDV-1 mutants M1, d1, d3, and M2, respectively.

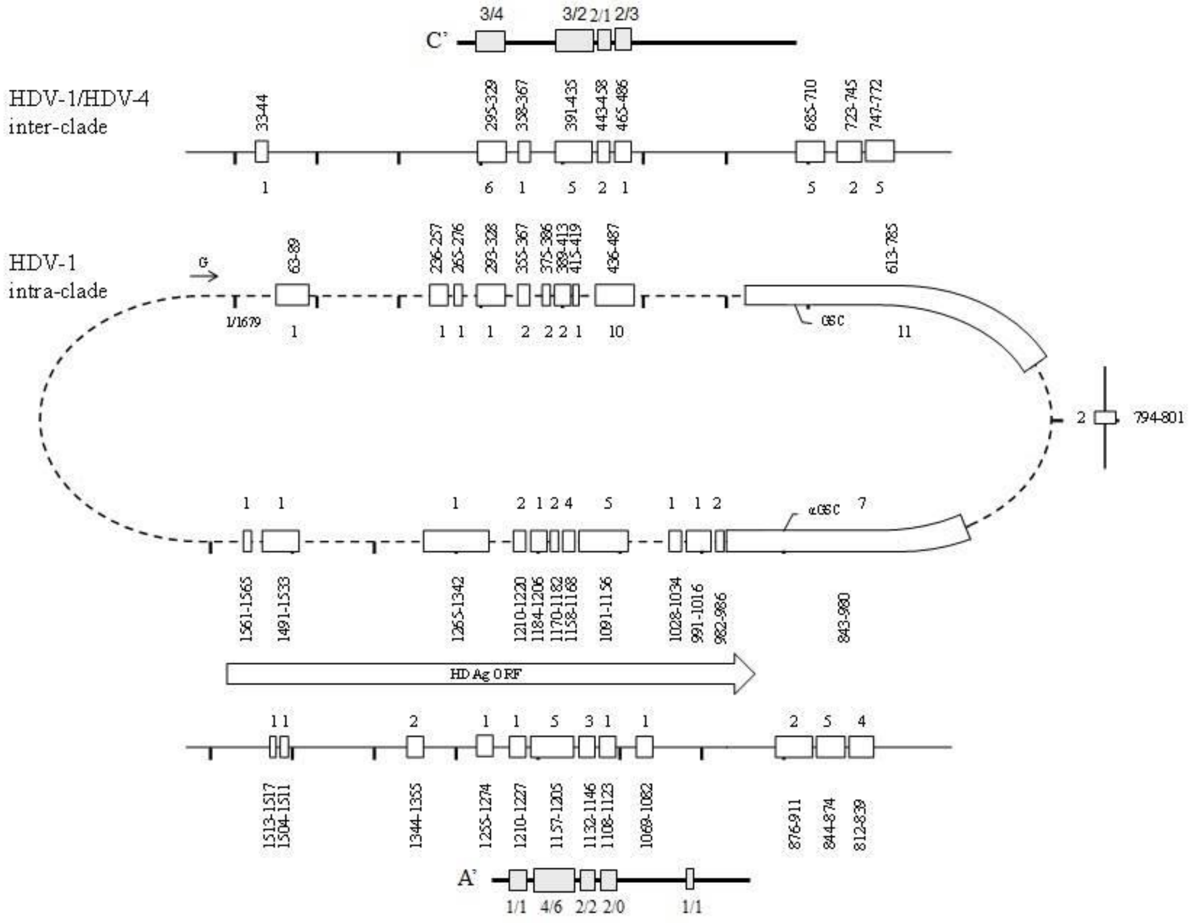

Supplementary Figure 5. Comparison of the crossovers in the intra- and inter-clade recombination maps. The HDV-1 intra-clade recombination map, which was adapted from a previous publication [28], is shown in the middle of the figure. The HDV rod-like RNA is represented by a black-outlined oval. The open boxes represent the crossovers. The HDV-1/HDV-4 inter-clade recombination map established in this report is represented as open boxes on three black lines, and is summarized outside the HDV-1 intra-clade recombination map. The nt numbers and clone numbers of the sequenced recombinants are shown.

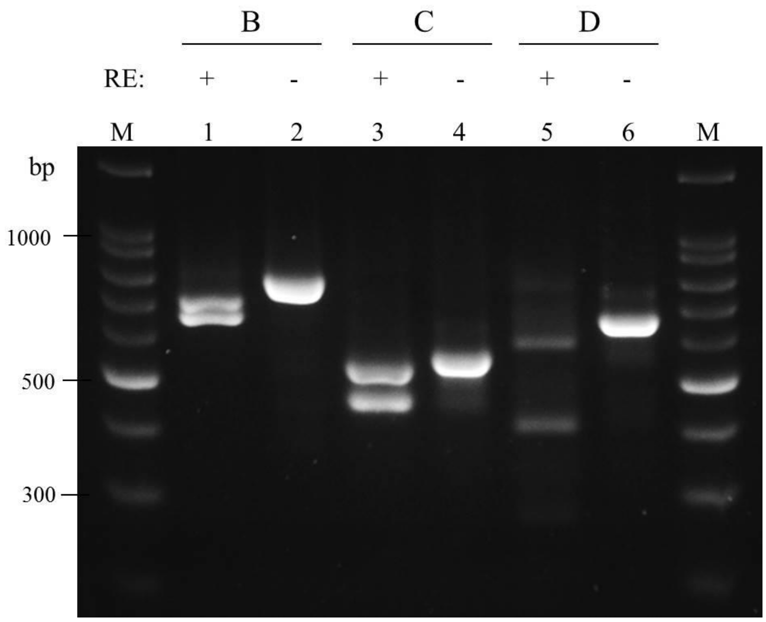

Supplementary Figure 6. Digested PCR products of mixed total cellular RNAs extracted from cells transfected separately with HDV-1 and HDV-4. Analyses and labels were as described in the legend to Figure 1B. Note that double-digested bands were undetectable and undigested bands were extremely faint or undetectable.

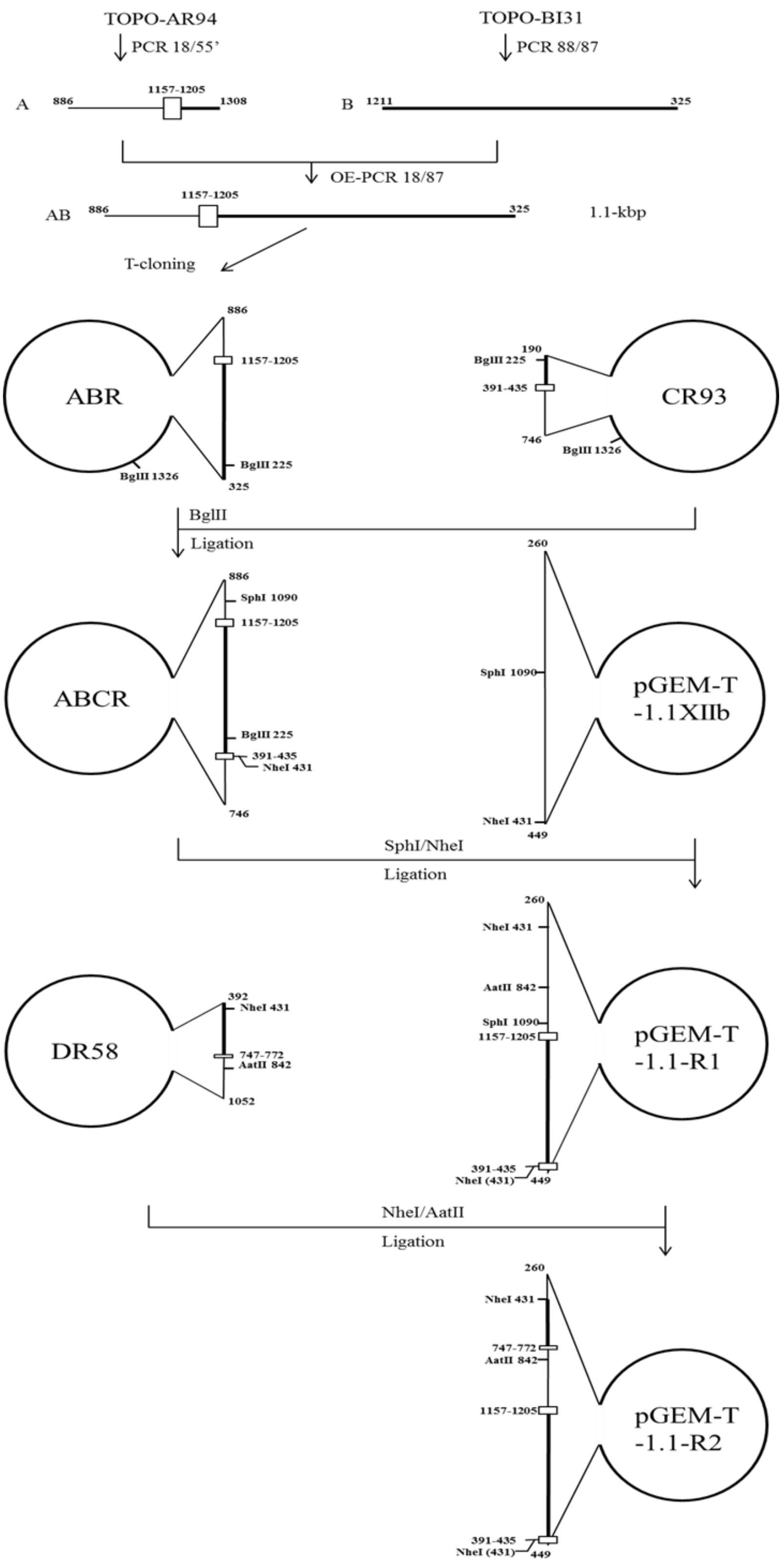

Supplementary Figure 7. Schematic for the construction of plasmids expressing the recombinant HDV R1 and R2 genomes. The HDV-1 and HDV-4 sequences are indicated by bold and thin lines, respectively. To assemble an HDV recombinant R1 genome with a

perfect rod-like structure, the inserts in TOPO-AR94 and TOPO-BI31 were PCR amplified using primer pairs 18/55’ and 88/87, respectively. The resulting PCR products were gel-purified (QIAquick Gel Extraction Kit, Qiagen) and joined by the PCR-based overlap extension method [65] using primers 18 and 87. The PCR-joined 1.1-kb fragment AB (nt 886-325) was gel-purified and cloned into a T-vector (TOPO PCR-II vector; Invitrogen), and the resulting plasmid was designated TOPO-ABR and confirmed by sequencing. The 3.9-kb and 1.5-kb bands produced from BglII-digested TOPO-ABR and TOPO-CR93, respectively, were gel-purified and ligated using a standard molecular cloning protocol [63]. The insert of the resulting plasmid, TOPO-ABCR, covered nt 886-746 and had recombination junctions located at nt 1157-1205 and nt 391-435. TOPO-ABCR was subjected to SphI and NheI double-digestion to release the 1-kb SphI(1090)-NheI(431) fragment of the HDV recombinant sequence. The SphI(1090)-NheI(431) fragment of HDV-4 from pGEM-T-1.1xIIb [20], which contained 1.1-mer (nt 260-449) of HDV-4 sequence, was removed by SphI and NheI double-digestion and replaced with the above-described SphI(1090)-NheI(431) fragment of HDV recombinant sequence excised from TOPO-ABCR. The resulting plasmid was designated pGEM-T-1.1xR1. To construct the plasmid expressing HDV recombinant genome R2, which had crossovers mapping to nt 747-772 and 1157-1205, the 0.4-kb NheI(431)-AatII(842) fragment was excised from pGEM-T-1.1xR1 and replaced with the NheI(431)-AatII(842) fragment released from NheI-AatII double-digested TOPO-DR58. The resulting plasmid was designated pGEM-T-1.1xR2.
