# Supplementary material for: Analyses of a whole-genome inter-clade recombination map of hepatitis delta virus suggest a host polymerase-driven and viral RNA structure-promoted template-switching mechanism for viral RNA recombination
Source: Oncotarget. 2017 Jun 1;8(37):60841–59. doi: 10.18632/oncotarget.18339 (PMC5617389; doi:10.18632/oncotarget.18339)

**Analyses of a whole-genome inter-clade recombination map of hepatitis delta virus suggest a host polymerase-driven and viral RNA structure-promoted template-switching mechanism for viral RNA recombination**

**Supplementary Tables**

**Supplementary Table 1.** **List of primers used for PCR amplification of the HDV genome**

| Primer name | Sequence (5’-3’)* | Location (nt) |
| --- | --- | --- |
| 88 | GTTCCTCTTCTTCCTCCCTGCTGAG | 1211-1235 |
| 87 | CGCTGAAGGGGTCCTCTGGAGGTG | 325-302 |
| 86 | GAGCGGGCCTCCCGATCCGAG | 190-210 |
| 74 | GACGGTCCCCTCGGAATGTTG | 746-726 |
| 80 | GAGACCGAAGCGAGGAGGAAAGC | 392-414 |
| 81 | GGGGACGGGCTGGACATCAGGGG | 1052-1030 |
| 77 | GAGATGCCATGCCGACCCGAAGAG | 883-906 |
| 78 | TAAGAGTTCTGAGGACCGCCGC | 1631-1610 |
| 138 | GAGAACAAGAAGAAGCAGC | 1274-1256 |
| 144 | GCTGCTTCTTCTTGTTCTCGAGGG | 1256-1279 |
| 48Ra | GATGTTCCCCAGCCAGGGATT | 1437-1457 |
| 48Rb | AATCCCTGGCTGGGGAACATC | 1457-1437 |
| 471-4f | aggcttatcccggggatcgg | 471-490 |
| 1045-4r | gcctggacaccaggggaact | 1045-1026 |
| 574-4f | AGCcgtgggaatccccagac | 574-593 |
| 730-1r | tgttgcccAgccggcgccAG | 730-711 |
| 686 G | aggctgggaccatgccggcc | 705-686 |
| 686 A | aggctgggaccatgccggct | 705-686 |
| 683 A | ctgggaccatgccggctatc | 702-683 |
| P-M1 | Cccaaccggcgggccggctattcttctttcccttctctcg | 1149-1188 |
| P-d1 | AAAGCAAAGAAAGCAA*-*GGGGCTAGCCGGTGGGTG | 410-444 |
| P-d3 | AAAGCAAAGAAAGCAA---GGCTAGCCGGTGGGTG | 410-444 |
| P-M2-1 | GCGAGGAGGAAAGCAAAGATAGCAACGGGGCTAGCCG | 401-437 |
| P-M2-2 | GCAAAGATAGCAACCGGGCTAGCCGGTGGG | 413-442 |
| F-1-1 | CTCCGGGACTCCCTGCAGAT | 1071-1090 |
| R-4-1 | CAAGAAGAAGCAGCTCTCCT | 1269-1250 |
| F-4-1 | ctccgggactcctagcatgc | 1071-1090 |
| R-1-1 | caagaagaagcagctatagg | 1269-1250 |
| 5’-1 | GCCCGAAGGGTTGAGTAGCACTCA | 243-266 |
| 3’-4 | GGGACCATGCCGGCCAAAGGTA | 700-679 |
| 5’-4 | GGCCCTAAGGTAGAATAGAACACC | 243-266 |
| 3’-1 | GGGACCATGCCGGCCATCAGGT | 700-679 |
|  |  |  |

*: “-” indicates a deletion and underline indicates a point mutation.

**Supplementary Table 2. Effect of the bulge-reducing mutants on distribution of the recombination junctions occurring at nt 243-700.**

| Recombination patterns | Recombination frequency (%) | | | | |
| --- | --- | --- | --- | --- | --- |
|  | Crossover regions (nt) | HDV-1 mutants used in co-transfections^^^ | | | |
|  |  | M1 | M2 | d1 | d3 |
| 5’-(1-4)-3’ 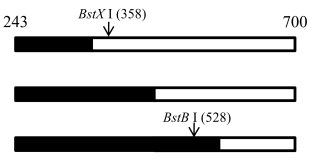 |  |  |  |  |  |
|  | 243-358 | 20.0 | 73.3 | 73.3 | 66.7 |
|  |  | 20.0 | 66.7 | - | - |
|  | 358-528 | 66.7 | 16.7 | 26.7 | 33.3 |
|  |  | 73.3 | 20.0 | - | - |
|  | 528-700 | 13.3 | 10.0 | 0 | 0 |
|  |  | 6.7 | 13.3 | - | - |
| 5’-(4-1)-3’ 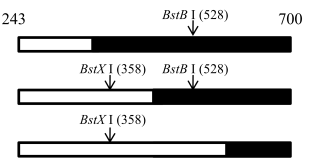 |  |  |  |  |  |
|  | 243-358 | 6.7 | 26.7 | 0 | 6.7 |
|  |  | 20.0 | 26.7 | - | - |
|  | 358-528 | 80.0 | 60.0 | 80.0 | 73.3 |
|  |  | 66.7 | 60.0 | - | - |
|  | 528-700 | 13.3 | 13.3 | 20.0 | 20.0 |
|  |  | 13.3 | 13.3 | - | - |

^^^: BstXI-BstBI-RFLP data are shown. The most frequent crossovers are shadowed.

**Supplementary Table 3.** **Templates and primers used to construct the HDAg-expressing plasmids**.


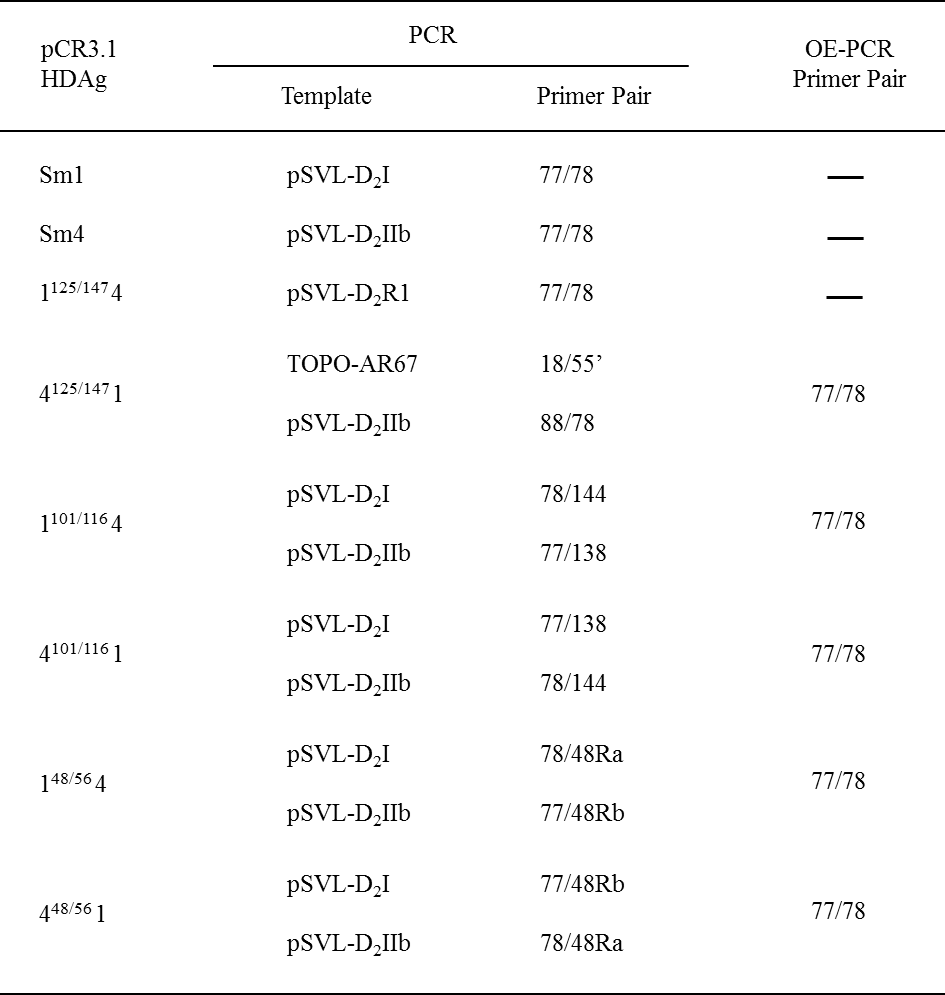

Supplement: Supplementary file 2 [file oncotarget-08-60841-s002.docx]
